# Brain structure–function associations identified in large-scale neuroimaging data

**DOI:** 10.1007/s00429-015-1177-6

**Published:** 2016-01-09

**Authors:** Zhi Yang, Jiang Qiu, Peipei Wang, Rui Liu, Xi-Nian Zuo

**Affiliations:** 1 Key Laboratory of Behavioral Science, Laboratory for Functional Connectome and Development and Magnetic Resonance Imaging Research Center, Institute of Psychology, Chinese Academy of Sciences, 16 Lincui Road, Chaoyang District, Beijing, 100101 China; 2 Shanghai Key Laboratory of Psychotic Disorders, Shanghai Mental Health Center, Shanghai Jiao Tong University School of Medicine, Beijing, 100101 China; 3 Faculty of Psychology, Southwest University, Chongqing, 400715 China; 4 Beijing Institute for Brain Disorders, Capital Medical University, Beijing, 100069 China; 5 Center for Higher Brain Function Research, School of Basic Medical Sciences, Capital Medical University, Beijing, 100069 China; 6 University of Chinese Academy of Sciences, Beijing, 100049 China; 7 Department of Psychology, School of Education Science, Guangxi Teachers Education University, Nanning, 530001 Guangxi China

**Keywords:** Structure–function association, Independent component analysis, Data mining, Connectomics, Multi-modal integration

## Abstract

The relationships between structural and functional measures of the human brain remain largely unknown. A majority of our limited knowledge regarding structure–function associations has been obtained through comparisons between specific groups of patients and healthy controls. Unfortunately, a direct and complete view of the associations across multiple structural and functional metrics in normal population is missing. We filled this gap by learning cross-individual co-variance among structural and functional measures using large-scale neuroimaging datasets. A discover-confirm scheme was applied to two independent samples (*N* = 184 and *N* = 340) of multi-modal neuroimaging datasets. A data mining tool, gRAICAR, was employed in the discover stage to generate quantitative and unbiased hypotheses of the co-variance among six functional and six structural imaging metrics. These hypotheses were validated using an independent dataset in the confirm stage. Fifteen multi-metric co-variance units, representing different co-variance relationships among the 12 metrics, were reliable across the two sets of neuroimaging datasets. The reliable co-variance units were summarized into a database, where users can select any location on the cortical map of any metric to examine the co-varying maps with the other 11 metrics. This database characterized the six functional metrics based on their co-variance with structural metrics, and provided a detailed reference to connect previous findings using different metrics and to predict maps of unexamined metrics. Gender, age, and handedness were associated to the co-variance units, and a sub-study of schizophrenia demonstrated the usefulness of the co-variance database.

**Electronic supplementary material:**

The online version of this article (doi:10.1007/s00429-015-1177-6) contains supplementary material, which is available to authorized users.

## Introduction

Many structural and functional metrics have been developed in neuroimaging studies, each characterizing the cerebral cortex from a different perspective. Brain structure metrics, such as cortical volume (vol), surface area (area), cortical thickness (thick), mean cortical curvature (curv), local gyrification index (lgi), and sulcus depth (sulc), have been derived from surface modeling of the cerebral cortex (Fischl et al. 1999) and widely applied to investigate brain development and brain disorders. For example, the vol, area, and thick metrics are popular measures used to quantify brain morphometric abnormalities in schizophrenia (e.g., Ohtani et al. 2014; Sprooten et al. 2013; Xiang et al. 2013). In particular, these metrics play different roles in distinguishing schizophrenia from bipolar disorder patients (Rimol et al. 2012). The curv, lgi, and sulc metrics have been developed to characterize gyri-folding patterns (Schaer et al. 2008), reflecting complex interactions between differential growth rates, various physical processes, and the specific layout of cortical fiber connections (White and Hilgetag 2011). These metrics have been proposed as potential candidates of effective brain-based markers for schizophrenia (e.g., Fornito et al. 2008; Nanda et al. 2014; Takahashi et al. 2013; White et al. 2003).

Similarly, a variety of functional metrics have been derived based on the temporal dynamics of blood oxygen level-dependent (BOLD) signals acquired under a task-free state (Biswal et al. 1995). Low-frequency fluctuations in task-free BOLD signal reflect intrinsic functional organizations of the brain (Biswal et al. 2010; Kelly et al. 2012; Yeo et al. 2011). In addition to traditional seed-based temporal correlations, metrics, including amplitude of low-frequency fluctuation (alff), degree centrality (dc), eigenvector centrality (ec), regional homogeneity (reho), and fractional amplitude of low-frequency fluctuation (falff), have been frequently used in functional imaging studies. Indeed, alff and falff metrics have been employed to quantify abnormalities in voxel-wise low-frequency fluctuations in schizophrenia patients (Yang et al. 2007; Zou et al. 2008; Zuo et al. 2010), and these metrics reflect distinguishable locations of abnormality in the cortex (Hoptman et al. 2010). The reho metric (Zang et al. 2004) reflects the local synchronization of low-frequency fluctuations as a local connectivity measure (for a comprehensive review, see Jiang and Zuo 2015). The use of the surface-based reho metric to exclude the influence of non-gray matter signals (i.e., partial volume effects) has recently been proposed (Zuo et al. 2013) in association with the hierarchical architecture of functional organization in the human brain (Jiang et al. 2015). The dc and ec metrics characterize the intrinsic functional organizations of the brain in a graph-theoretical perspective (Buckner et al. 2009; Bullmore and Sporns 2009; Lohmann et al. 2010; Zuo et al. 2012; Zuo and Xing 2014), which has been adopted in neuroimaging studies of schizophrenia to investigate deficits in brain network hubs (Alexander-Bloch 2010; Lord et al. 2011; for a review see Rubinov and Bullmore 2013).

While a number of studies have demonstrated superior performance of combining information from multiple metrics (or imaging modalities) to examine neural mechanisms underlying behaviors and distinguish mental disorders (Fan et al. 2008; Kim et al. 2012; Palaniyappan and Liddle 2014; Wee et al. 2012; Zhang et al. 2011a), the relationships between these structural and functional metrics are largely unknown. Specifically, our understanding of the associations across multiple neuroimaging metrics is restricted in three aspects: first, nearly all multi-modal studies integrate no more than three metrics; second, many studies combine information from multiple metrics based on their spatial overlaps, but relevant metrics do not necessarily overlap in space; and third, most studies compare specific groups of patients to healthy controls. Due to these practical issues, we currently only have indirect knowledge about the similarities and differences across these metrics (Sui et al. 2014), and a direct/complete view of the associations across multiple structural and functional metrics in normal population is lacking.

To map the cross-subject co-variance between the aforementioned structural and functional metrics, we applied a discover-confirm scheme to two independent large samples (*N* = 184 and *N* = 340) of multi-modal neuroimaging datasets. In the discover stage, we used the first sample and a data-driven approach, generalized ranking and averaging independent component analysis (gRAICAR^1^; Yang et al. 2008, 2012) to generate quantitative hypotheses about the cross-subject co-variance among the 12 metrics. In the confirm stage, we examined these hypotheses using the second dataset. This set of analyses is able to address the practical difficulties mentioned above because (1) this data-driven approach generates unbiased hypotheses (imposes no constraint of spatial similarity between metrics), (2) the discover-confirm scheme is robust to artifacts introduced by odd images, and (3) the model is simple enough to include a number of metrics. We hypothesize that the co-variance relationships among the 12 metrics, represented by multi-metric co-variance units (MMCUs), are reliable and reproducible across datasets, and that these MMCUs are able to interpret variation in behavioral variables, to link existing findings using different metrics, and to predict characteristics of unexamined metrics for individual studies.

## Methods

### Datasets

We used two publicly available large datasets from the 1000 Functional Connectomes Project (FCP; http://fcon_1000.projects.nitrc.org/fcpClassic/FcpTable.html) (Biswal et al. 2010) and the Consortium for Reliability and Reproducibility (Zuo et al. 2014) (CoRR; http://fcon_1000.projects.nitrc.org/indi/CoRR). Dataset 1 is from the FCP, the Cambridge-Buckner site (FCP-Cambridge), where high-resolution structural images and resting-state functional images were collected from 198 healthy subjects (123 females, ages: 18–40) using a Siemens 3 T Trio scanner. The imaging sequences included MP-RAGE for T1 image acquisition (TR/TE/TI = 2200/1.04–7.01/1100 ms, Flip Angle = 7°, FOV = 230 mm, 144 sagittal slices, voxel size = 1.2 × 1.2 × 1.2 mm) and EPI for resting-state functional MRI (rfMRI) image acquisition (TR/TE = 3000/30 ms, FA = 85°, FOV = 216 mm, 47 axial slices, voxel size = 3.0 × 3.0 × 3.0 mm, 124 volumes).

Dataset 2 is a part of the Consortium for Reliability and Reproducibility (CoRR; Zuo et al. 2014) and contains 345 healthy college students (182 females; ages: 17–27) recruited from Southwest University in Chongqing, China (CoRR-SWU). Each participant underwent both MP-RAGE (TR/TE/TI = 1900/2.52/900 ms, Flip Angle = 9°, FOV = 256 mm, 176 sagittal slices, voxel size = 1.0 × 1.0 × 1.0 mm) and rfMRI scanning (TR/TE = 2000/30 ms, FA = 90°, FOV = 220 mm, 32 axial slices, voxel size = 3.44 × 3.44 × 4.0 mm, 242 volumes) using a Siemens 3 T Trio scanner.

### Preprocessing and quality control: structural images

All structural MRI images were first inspected for quality control before being preprocessed using the structural preprocessing pipeline of the Connectome Computation System (CCS: Xu et al. 2015). This pipeline first cleaned the images with a spatially adaptive non-local means filter and corrected the intensity inhomogeneity. CCS then called FreeSurfer (Dale et al. 1999) to implement the steps of brain tissue segmentation and cortical surface reconstruction. Specific steps included: (1) brain extraction with a hybrid watershed/surface deformation; (2) tissue segmentation of the cerebrospinal fluid (CSF), white matter (WM) and deep gray matter (GM); (3) cutting plane generation to disconnect the two hemispheres and subcortical structures; (4) fixation of the interior holes of the segmentation; (5) a triangular mesh tessellation over a GM-WM boundary and mesh deformation to produce smooth GM-WM (white surface) and GM-CSF interfaces (pial surface); (6) topological defect correction on the surface; (7) individual surface mesh inflation to a sphere; and (8) estimation of the deformation between the resulting spherical mesh and a common spherical coordinate system to align the cortical folding patterns across subjects. As a quality assessment, intermediate results from the above steps were visually inspected based upon the screenshots generated using CCS. Subjects with bad brain extraction, tissue segmentation and surface reconstruction were excluded from the subsequent analysis.

For each subject, six morphological metrics were derived from the cortical surface model created using FreeSurfer, including cortical vol, area, thick, curv, lgi, and sulc. All abbreviations are listed in Supplementary Text 1, and detailed descriptions of the meaning and computation of these metrics are presented in Supplementary Text 2. The area metric was set to the total area of the triangles connected to a vertex (Fischl and Dale 2000). The total cortical area generated using the FreeSurfer model was consistent with the surface area derived from postmortem studies and has been validated on several brain phantoms and compared with other surface-based analysis packages (Lee et al. 2006; Makris et al. 2006). The thick metric was calculated using the average linking distance between the pial and white surfaces (Fischl and Dale 2000). This measure of cortical thickness showed adequate test–retest reliability across time, scanner manufacturers and field strengths (Han et al. 2006). The vol metric was calculated as the product of the thick and area metrics. The curv metric represents the mean of the two principal curvatures, which measures the maximum and minimum bending of the cortical surface at the vertex (Pienaar et al. 2008). The lgi is a metric that quantifies the amount of cortex buried within the sulcal folds compared with the amount of cortex on the outer visible cortex. A cortex with extensive folding has a large lgi, whereas a cortex with limited folding has a small lgi. In Freesurfer, lgi was computed at thousands of points over the entire cortical surface (Schaer et al. 2008). Moreover, the sulc metric is the integrated dot product of the movement vector with the surface norm during inflation, indicating the large-scale geometry of the cortical surface. When deep regions consistently move outward, these regions have positive values of sulc, whereas when superficial regions move inward, these regions have negative values.

Each of these metrics was computed in the native space and subsequently transferred onto the *fsaverage* standard spherical surface. To enable further comparisons with functional metrics, these metric maps were down sampled to an *fsaverage5* standard cortical surface with 10,242 vertices per hemisphere, and a Gaussian filter (FWHM = 4 mm) was applied to smooth the surface maps. An outlier detection procedure was further performed to exclude subjects with extremely low/high metrics. A subject was excluded from subsequent analyses when any of the six metrics was an outlier at any vertex of the *fsaverage5* surface. The outliers were determined using the generalized extreme Studentized deviate test (*p* < 0.001) (Rosner 1983).

### Preprocessing and quality control: functional images

Connectome computation system was employed to analyze rfMRI data using a functional pipeline. The following procedures were included: (1) eliminating the first 5 EPI volumes from each scan for signal equilibration; (2) de-spiking the time series to detect and reduce outliers (spikes) using an hyperbolic tangent function; (3) slice timing using Fourier interpolation to temporally correct the interleaved slice acquisition; (4) aligning each volume to a “base” image (the mean EPI image) using Fourier interpolation to correct between-head movements; (5) normalizing the 4D global mean intensity to 10,000 to allow inter-subject comparisons; (6) regressing out the WM/CSF mean time series and the Friston-24 motion time series to reduce the effects of these confounding factors; (7) filtering the residual time series with a band-pass filter (0.01–0.1 Hz) to extract low-frequency fluctuations; (8) removing both linear and quadratic trends; and (9) aligning individual motion-corrected functional images to the individual structural image using a GM-WM boundary-based registration (BBR) algorithm. The preprocessed time series were subsequently projected onto the *fsaverage5* standard cortical surface.

For each subject in both preprocessed datasets, we computed six functional metrics that character low-frequency fluctuations (0.01–0.1 Hz), including alff and its fractional variant (falff), dc, ec, and surface-based regional homogeneity with length-one (reho) and length-two (reho2) neighbors. These metrics were commonly used to characterize properties of intrinsic connectivity networks (see Zuo and Xing 2014 for a review). Here, we provide a brief description of these metrics and more details are presented in Supplementary Text 3. The alff metric indicates the power of low-frequency oscillations in the BOLD time series (Zang et al. 2007). The normalized form, falff, further divides the alff value by the power of the entire frequency spectrum (Zou et al. 2008). These metrics characterize single voxel/vertex properties. In contrast, from a graph-theoretical perspective (Bullmore and Sporns 2009), the dc and ec metrics depict the relationships of the BOLD time series between a given location and all other locations in the brain. The relationships between the time series were characterized using Pearson’s Correlation coefficients. The reho and reho2 metrics measure the multi-vertex relationship in a local neighborhood (Zang et al. 2004; Zuo et al. 2013), reflecting the local homogeneity of the BOLD time series. The radius of the local neighborhood is different between the reho and reho2 metrics, where the reho metric has a radius of 1 vertex, and reho2 has a radius of 2 vertices.

We conducted the following quality assessments for functional images. To examine the head motion during rfMRI acquisition, we computed (1) the maximum distance of translational head movement (maxTran), (2) the maximum degree of rotational head movement (maxRot), and (3) the mean frame-wise displacement (meanFD). To evaluate the quality of functional–structural image realignment with BBR registration, we collected the minimal cost of the BBR co-registration (mcBBR). Subjects were excluded from further analysis when the following criteria were not met: (1) maxTran ≤ 2 mm, (2) maxRot ≤ 2°, (3) meanFD ≤ 0.2 mm, and (4) mcBBR ≤ 0.75. As a result of the quality control procedures for both structural and functional images, 184 subjects from FCP-Cambridge (female 117, age range 18–30) and 340 subjects from CoRR-SWU (female = 180, age range 17–27) were retained for further analyses.

### Structure–function co-variance analysis: discovery

**Fig. 1:**
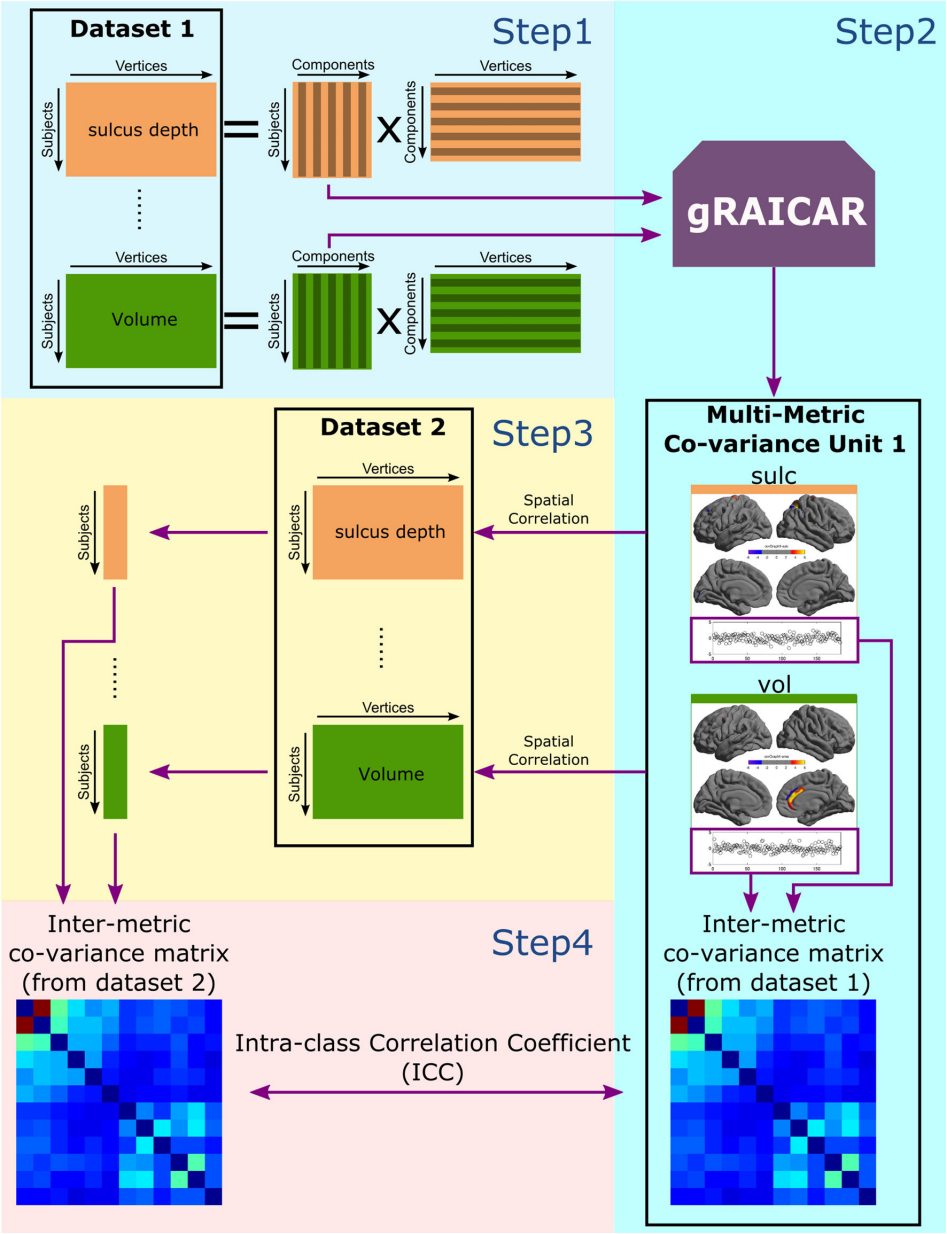
Flowchart of the discover-confirm scheme. For an animated demonstration, see http://www.ddl.escience.cn/f/qMsE. In the discover stage, the six structural metrics (vol, area, thick, curv, lgi, sulc) and six functional metrics (alff, falff, dc, ec, reho, reho2) were derived from each subject in FCP-Cambridge (*N* = 184). Each of the 12 metrics was decomposed into spatial components using independent component analysis (*step1*). gRAICAR was used to pool the subject courses of the resulting components from all 12 metrics and align the components to form multi-metric co-variance units (MMCUs, *step2*). Each MMCU unit associates the surface component maps (SCMs) from different metrics according to the co-variance across subjects (correlation between corresponding subject courses). The levels of co-variance between pairs of SCMs are depicted in the inter-metric co-variance matrix. In the confirm stage, spatial correlations were computed between the SCMs identified in FCP-Cambridge and the metric maps independently derived from CoRR-SWU (*N* = 340), yielding a subject course for each SCM (*step3*). The correlations between the subject courses were computed to generate a new inter-metric co-variance matrix. For each MMCU, the similarity of the two inter-metric co-variance matrices, derived from FCP-Cambridge and CoRR-SWU, respectively, were quantified using intra-class correlation coefficients (ICC) that indicate the consistency of the matrices across the two datasets, relative to the similarity between the matrices from different MMCUs

A discover-confirm scheme was illustrated in Fig. 1 and was applied to the preprocessed FCP-Cambridge (*N* = 184) and CoRR-SWU (*N* = 340) datasets. Besides Fig. 1, we made an animation to demonstrate the procedures.^2^ FCP-Cambridge was used in the discover stage. We first decomposed the cross-subject variability of each cortical metric in FCP-Cambridge into a number of independent components. Specifically, an *n* by *v* matrix was formed for each of the 12 metrics, where *n* is the number of subjects (*n* = 184), and *v* is the number of vertices on the surface map (for the ‘fsaverage5’ mesh, *v* = 20,484). Independent component analysis (ICA), as implemented in the MELODIC module of FSL software (Beckmann and Smith 2004), was applied to the matrices (step 1 in Fig. 1). The numbers of components were automatically determined using the Laplacian estimator in MELODIC. The ICA outputs are referred to as surface component maps (SCMs), each representing a spatial pattern of vertices. Each SCM is accompanied with a subject course that depicted the variation of the corresponding SCM across subjects. Since the SCM is an unthresholded, continuous map of vertices, the values on the vertices indicate the relative contribution of the vertices to the subject course. The above ICA decomposition was applied to the 12 metrics separately.

Next, we adopted gRAICAR (Yang et al. 2008; Yang et al. 2012) to cluster the SCMs from the 12 metrics (step 2 in Fig. 1). The gRAICAR algorithm is effective in revealing intrinsic inter-subject relationships (Kyathanahally et al. 2015; Yang et al. 2014a) or patient subtypes (Yang et al. 2014b). Supplementary Fig. 1 presents a demonstration of the gRAICAR algorithm. Specifically, gRAICAR first constructed a full similarity matrix (FSM) containing pair-wise similarity between all the SCMs from the 12 metrics (Fig. S1A). The similarity between the SCMs was defined as the Pearson’s correlation coefficients between their subject courses. The value of this similarity reflects the coherence between two SCMs in terms of their cross-subject variations. gRAICAR then searched through the FSM for clusters of SCMs (demonstrated in Fig. S1). The first operation in the search procedure was ranking the SCMs according to their “popularity” among SCMs from different metrics. The following steps were conducted to compute the “popularity” of SCMs: (1) the FSM was segmented into metric-blocks, so that the FSMs from the same metric stayed together and the similarity values between them were represented in a block within the FSM (Fig. S1A). (2) The similarity values were converted into relative values (Zrow) within each row in each metric-block (Fig. S1B). (3) The maximal value in each row of each metric-block in Zrow was then retained in a new matrix (Zmax), and all other values were set to 0 (Fig. S1C). Each row in the Zmax matrix thus revealed the most similar SCMs from different metrics to the SCM represented in current row. (4) The Zmax matrix was multiplied to its transform (multiply elements in corresponding locations, Fig. S1D). This operation eliminated unpaired inter-metric maximal similarities. (5) In the resultant matrix, the sum of each row yielded the “popularity” value for each SCM, representing a special form of centrality that takes specificity and mutual correspondence of SCMs into account. We then computed a standardized FSM by adding the Zrow matrix to its transpose, so that the similarity between a pair of SCMs from different metrics was standardized among all SCMs from the relevant metrics (Fig. S1E). Starting from the top SCM in the popularity rank, 11 SCMs from different metrics were identified in the standardized FSM, by searching for maxima in metric-blocks on the top SCM’s row (Fig. S1F).

As a result, the identified 12 SCMs shared similar trends of variability across subjects, and were grouped as a multi-metric co-variance unit (MMCU). An inter-metric co-variance matrix was extracted from the FSM for each MMCU to characterize the subject course similarity between SCMs from different metrics (Fig. S1G). These 12 SCMs were thus eliminated from the popularity rank, and the above procedure was repeated until all SCMs were assigned to MMCUs. Because different metrics had different numbers of SCMs, the number of MMCUs detected using gRAICAR equals the second maximal number of SCMs among the 12 metrics (An MMCU at least associates two metrics).

### Structure–function co-variance analysis: validation

In the confirm stage, we examined whether the MMCUs detected in FCP-Cambridge were reproducible across datasets. This step is necessary because odd subjects or image artifacts might influence the MMCUs obtained in FCP-Cambridge. We first correlated each SCM in each MMCU (obtained in FCP-Cambridge) to the corresponding metric map of each subject in CoRR-SWU. As a result, we obtained a series of correlation coefficients; each coefficient was from a participant in CoRR-SWU. We treated the correlation coefficients as a cross-subject fluctuation course in CoRR-SWU for a given SCM in a given MMCU obtained in FCP-Cambridge (step 3 in Fig. 1). For instance, we correlated the SCM of the vol metric (obtained in FCP-Cambridge) to the vol map of all subjects in CoRR-SWU, yielding a course of correlation coefficients that represented fluctuating contributions of the vol SCM to the subjects in CoRR-SWU.

Next, for each MMCU, we computed a correlation matrix between the cross-subject fluctuation courses of the 12 member SCMs in CoRR-SWU. We then used the intra-class correlation coefficient (ICC) to measure the consistency between the inter-metric co-variance matrices obtained in the CoRR-SWU and FCP-Cambridge datasets (step 4 in Fig. 1). The rationale for using ICC here is to consider both the intra-MMCU variability and the inter-MMCU variability. In this step, the elements of the co-variance matrices were first converted into Fisher’s Z scores, and the co-variance matrices obtained in both datasets were deemed as two observations of the same variable. The resultant ICC, therefore, indicates the consistency between the inter-metric co-variance matrices obtained in FCP-Cambridge and CoRR-SWU, relative to the variability/similarity between the inter-metric co-variance matrices from different MMCUs.

We conducted a permutation test to examine the significance of the ICC values. Specifically, the SCMs from different metrics were randomly grouped into fake MMCUs, and inter-metric co-variance matrices were computed by correlating the SCMs’ subject courses in FCP-Cambridge. Meanwhile, the subject courses corresponding to these SCMs were obtained in CoRR-SWU and were correlated to yield inter-metric co-variance matrices in CoRR-SWU. An ICC was then computed between every pair of fake inter-metric co-variance matrices (from FCP-Cambridge and CoRR-SWU, respectively). This procedure was repeated 5000 times to generate a null distribution of ICCs. Percentiles corresponding to the original ICCs were calculated in this null distribution, yielding empirical significance levels. This permutation test was only applied to MMCUs with ICC values above 0.4, since 0.4 was commonly deemed as a threshold for fair reproducibility.

### Examine relevance to demographical variables

The MMCUs with significant reproducibility were further examined for their relevance to demographical variables including age (18–30), sex (117 females), and handedness (25 left handed). Specifically, for each MMCU, the subject courses (obtained from ICA in the discover stage) corresponding to the member SCMs were collected and input into a principle component analysis. The first principle component represented the most common variance across all the subject courses and thus can be considered as a representative subject course for the given MMCU. A regression model was then constructed to explain this representative subject course by variability in age, sex, and handedness.

## Results

### Discover MMCUs from FCP-Cambridge data

In FCP-Cambridge, we decomposed cross-subject variability for each metric into a number of components. The numbers of components were automatically estimated using the Laplacian approach implemented in MELODIC. As a result, different metrics were decomposed into a different number of components. The numbers of components for the 12 metrics estimated in the FCP-Cambridge dataset are summarized in Supplementary Table S1. Each metric component carries two pieces of information: a surface map (i.e., SCM) reflecting the spatial distribution of a given metric, and a subject course indicating the variable contributions from different subjects to the current component.

The SCMs decomposed from different metrics in the FCP-Cambridge dataset were clustered into MMCUs according to the similarity of their associated subject courses. gRAICAR revealed 88 MMCUs that reflect co-variance of at least two SCMs (see Fig. 3 for an example of an MMCU). A typical MMCU collects 12 SCMs that exhibit similar subject courses, each from a different metric. To characterize the co-variance relationships among the 12 metrics, each MMCU is associated with an inter-metric co-variance matrix that shows correlation coefficients between every pair of SCMs. Notably, because the cross-subject variability of different metrics was decomposed into different numbers of SCMs, it is not guaranteed that each MMCU includes SCMs from all 12 metrics.

### Validate reliable MMCUs using CoRR-SWU data

The MMCUs obtained in FCP-Cambridge represent common cross-subject variability among metrics. Before interpreting the neurobiological meaning of these MMCUs, the reliability of these MMCUs should be rigorously examined. In other words, the MMCUs obtained in FCP-Cambridge might represent impact from odd subjects or image artifacts. To exclude this possibility, we used CoRR-SWU to identify reliable MMCUs across datasets. Reliability was measured based on the ICC between the inter-metric co-variance matrices obtained in FCP-Cambridge and CoRR-SWU. Among the 88 MMCUs detected in FCP-Cambridge, we identified 42 MMCUs with ICC values greater than 0.40. This threshold is typically considered as a cutoff for fair reproducibility (Rosner 1995). These 42 MMCUs likely reflected reliable (not caused by odd subjects or image artifacts) co-variance between different metrics. Among these, 15 MMCUs had ICC values greater than the 95.5th percentile of the null distribution of ICCs (ICC >0.81), they were considered as significantly reliable at *p* < 0.005. These MMCUs are presented in Supplementary Figures S2–S16.

We made a database with an interactive graphic user interface to present all the 42 MMCUs (https://yangzhi.shinyapps.io/showCovGraph_R). Figures 3 and 4 below are screen captures of the user interface. Users can click on any location of the 12 metric maps, and the interface will render the co-variance relationships between all surface maps to show the MMCU maximally affected by the current location. As users’ mouse hover over the surface maps, the texts in the upper right corner will simultaneously display the index and ICC value of the most dominate MMCU at the location under the mouse. This database provides an efficient way to explore the co-varying maps across all metrics.

### General co-variance relationships across 12 metrics

**Fig. 2:**
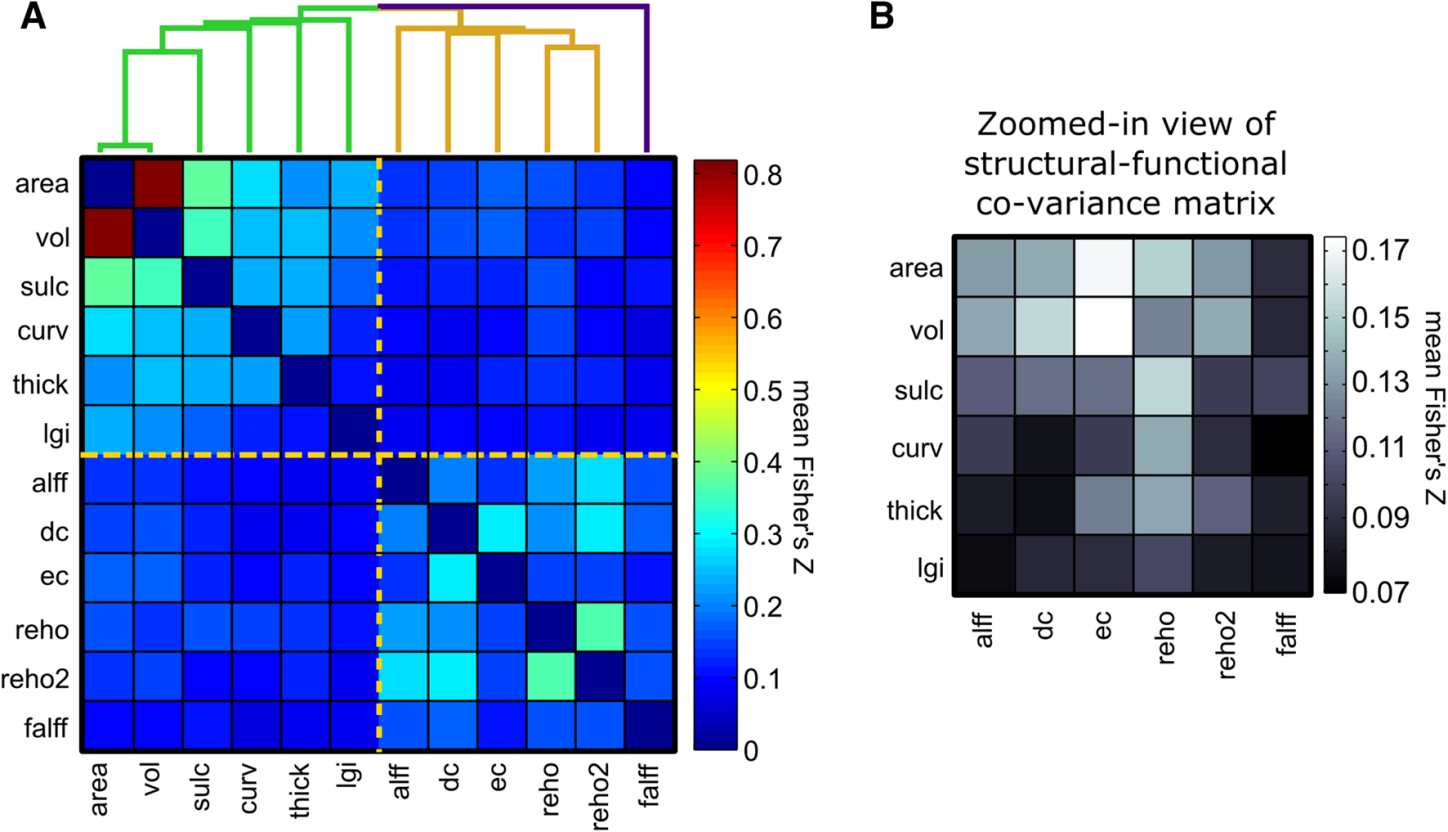
Overall co-variance among the 12 structural and functional metrics. **a** General similarity matrix obtained after averaging the inter-metric co-variance matrices from the 42 MMCUs (transformed to Fisher’s *Z* scores before averaging). The *colors* indicate the mean Fisher’s *Z* scores. A higher *Z* score indicates higher general co-variance between the two corresponding metrics. The *dashed lines* over the matrix separate structural and functional metrics. The hierarchical clustering dendrogram shows that the structural and functional metrics belong to two clusters, except for the falff metric. **b** A zoomed-in view of the general structural–functional co-variance matrix. The *color scales* are adjusted to enhance the contrast. The ec metric (functional) shows the maximal general co-variance with the area and vol metrics (structural)

To summarize the general co-variance relationships across the 12 metrics, we averaged the inter-metric co-variance matrices of all 42 MMCUs (first transformed each element in the matrices to Fisher’s *Z* score), yielding an overall inter-metric co-variance matrix (see Fig. 2a). This matrix reflected the degree of cross-subject co-variance between every pair of metrics, despite the specific locations shown in the SCMs. Using this matrix, we can infer whether two metrics carry common variability information. Overall, the co-variance among the 12 metrics was generally low (mean correlation coefficients 0.170, SD 0.118), but significantly larger than 0 [*t*(65) = 8.820, *p* < 0.0001]. The general co-variance between the functional and structural metrics was even lower (mean correlation coefficients 0.112, SD 0.03), but significantly larger than 0 [*t*(35) = 22.25, *p* < 0.0001]. The correlation coefficients among the six structural metrics were significantly larger than those between the structural and functional metrics [*t*(14.13) = 2.88, *p* = 0.012]. Similarly, the correlation coefficients among the six functional metrics were also significantly larger than those between the structural and functional metrics [*t*(15.96) = 3.87, *p* = 0.0014].

Hierarchical clustering analysis on this matrix showed that the six functional metrics carry different information from the structural metrics. As Fig. 2a shows, the vol and area metrics exhibited a high degree of co-variance, whereas the other structural metrics showed some independence from the vol and area metrics. The co-variance between the six functional metrics was not as strong as the structural metrics. Nonetheless, the reho2 metric showed relatively strong co-variance with multiple metrics, including alff, dc, and reho. The dc metric showed relatively strong co-variance with ec. The falff metric might represent a special functional metric that shares little co-variance with the other functional and structural metrics.

To take a closer look into the co-variance between functional and structural metrics, a zoomed-in view of the structural–functional co-variance is shown in Fig. 2b, located in the upper right quadrant of the matrix shown in Fig. 2a. Figure 2b suggests that the functional metric ec has the highest co-variance with the structural metrics area and vol (*r* = 0.309). The functional metric reho co-varied with multiple structural metrics, including area, sulc, curv, and thick (*r* = 0.159, 0.154, 0.138, and 0.135, respectively). Among these structural metrics, curv and thick showed specificity in co-variance with reho. Interestingly, reho2 exhibited a different co-variance pattern with structural metrics than reho, but more similar to that of ec. The difference between the within-structural and within-functional correlations was not significant [*t*(15.88) = −1.79, *p* = 0.093].

### Structural–functional MMCUs

**Fig. 3:**
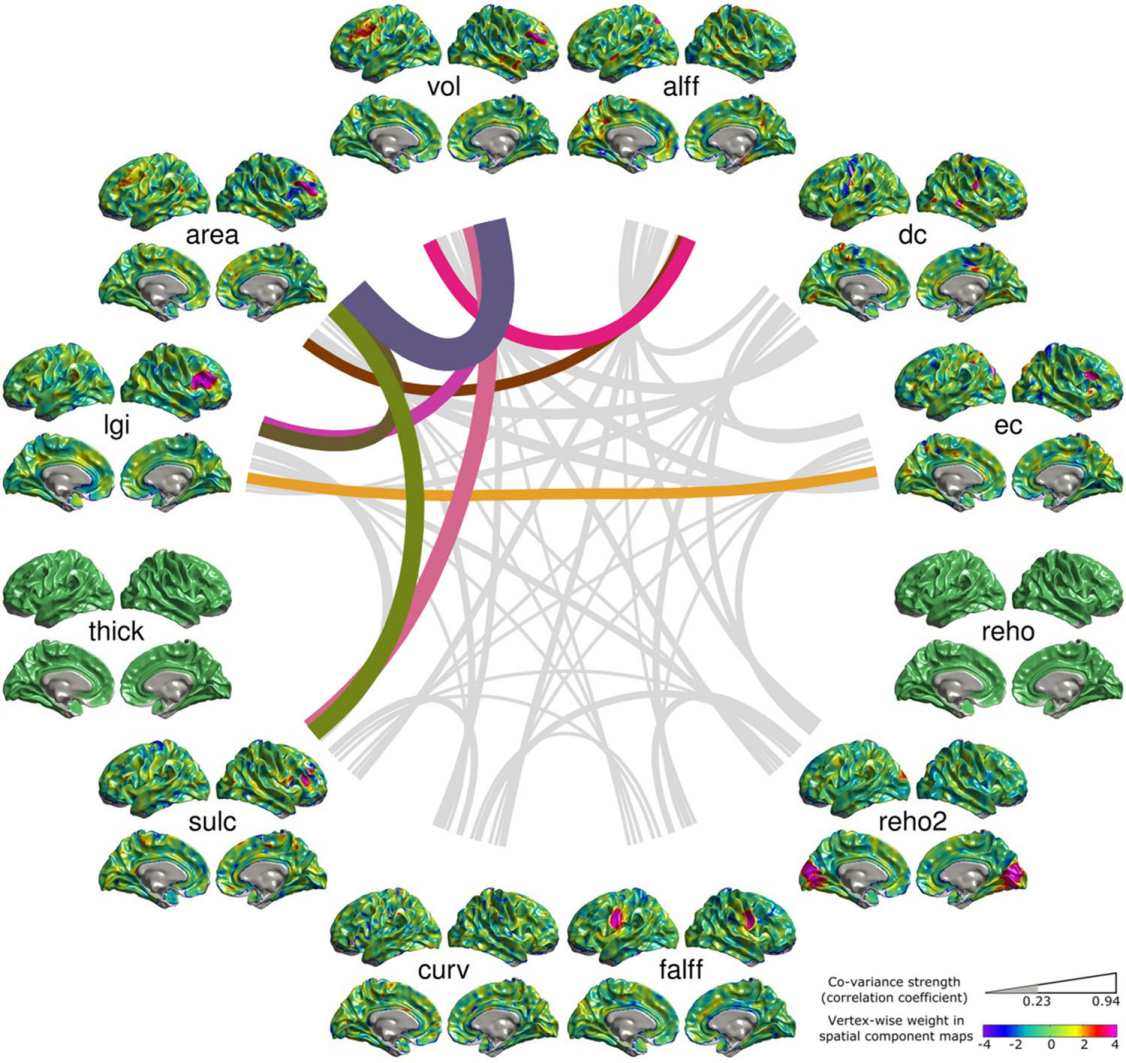
The structural–functional co-variance unit with the highest ICC value (ICC = 0.78, *p* = 0.0022). The SCMs are arranged on a* circle*. Abbreviations of the metrics are labeled at the* center* of each SCM. The weights of the vertices (*Z* scores) on the SCMs are indicated using *colors* (see *color bar* at the *bottom-right* corner). The SCMs are linked using curves whose width indicates the correlation coefficients between the subject courses of the SCMs. Only the statistically significant links are rendered using* colors* other than* gray* (*r* > 0.23, *p* < 0.0005, multiple comparison error corrected using the Bonferroni approach). This multi-metric co-variance unit shows significant structure–function co-variance between the ec and lgi SCMs and between the alff, area, and vol SCMs

Among the 15 significantly reliable MMCUs, 5 MMCUs (Figs. S9, S10, S11, and S15) showed significant co-variance between structural and functional metrics (*r* > 0.23, *p* < 0.0005). Figure 3 presents the structural–functional MMCU with the highest ICC (ICC = 0.83, *p* = 0.0025). This figure shows significant co-variance between the ec and lgi SCMs and between the alff, area, and vol SCMs. A remarkable commonality across the lgi, area, vol, and ec SCMs is the cross-subject variability around the right superior frontal gyrus (SFG). The alff SCM shows noticeable cross-subject variability on the precuneus, posterior cingulate, left dorsal prefrontal cortex, and lateral and inferior temporal lobe. The strong co-variance in this MMCU suggests that the structural variability in the right SFG predicts ec of this region as well as alff of remote regions such as the precuneus and posterior cingulate cortex.

### Relevance to demographical variables

**Fig. 4:**
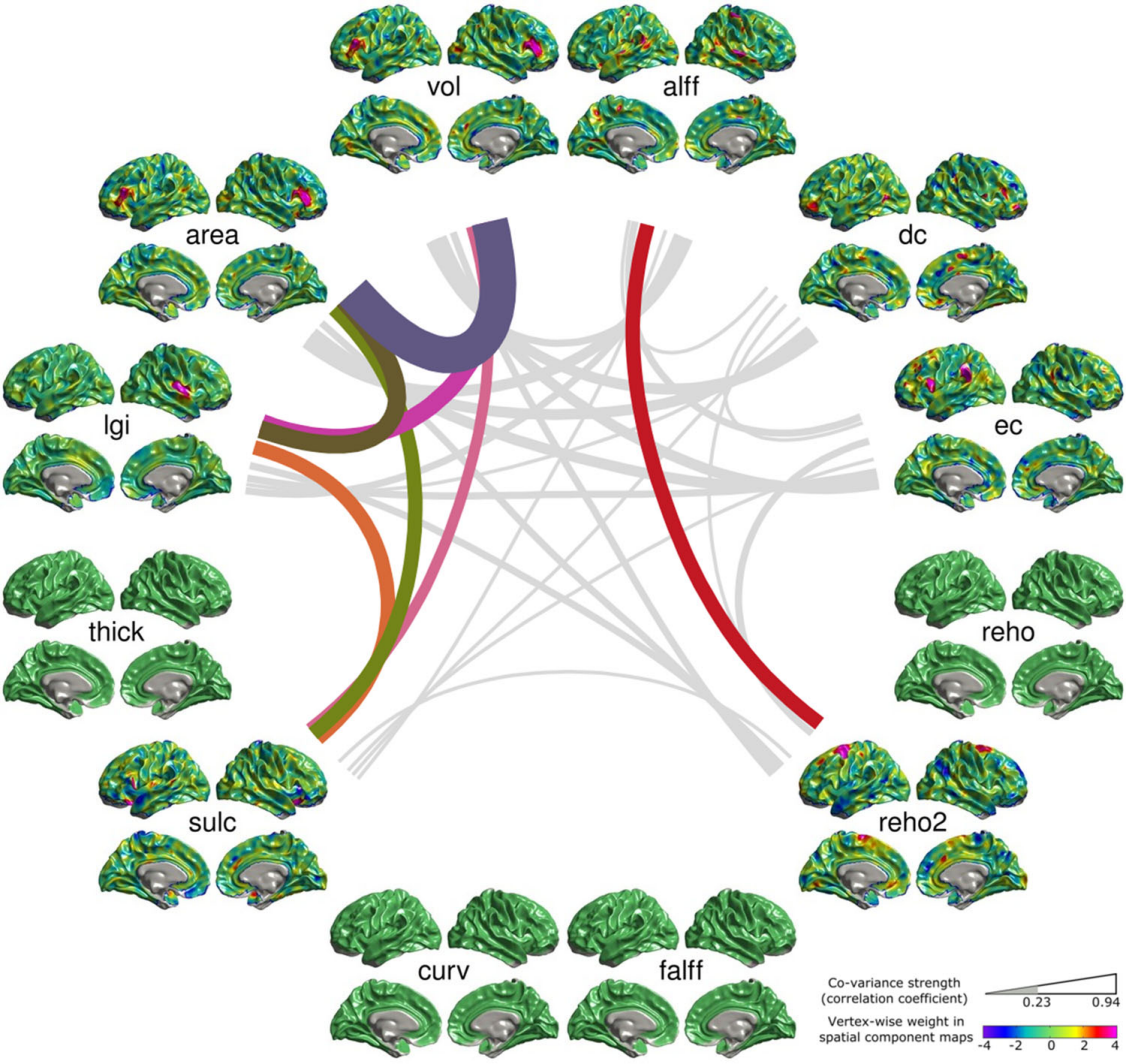
The structural–functional co-variance unit showing significant sex and handedness effects. For a complete description of this demonstration, see the legend of Fig. 3. This MMCU indicates co-variance among the vol and area of the middle frontal gyrus, the lgi of the right STG, the sulc of the inferior frontal gyrus, the alff of the bilateral STG and the right post-central gyrus, and the reho2 of the STG

We examined the linear relationships between the MMCUs’ representative subject courses and the demographical variables including age, sex, and handedness. Among the 15 MMCUs, four showed significant sex effect after Bonferroni’s correction (Figs. S6, S8, S13, and S14, *p* < 0.0005), and one of them (Fig. S13) showed significant handedness effect (*p* = 0.002). No MMCU exhibited significant age effect after Bonferroni’s correction. Figure 4 presents the MMCU showing both significant sex (*p* = 7.5 × 10^−7^) and handedness effects (*p* = 0.002). In this MMCU, the vol and area of the middle frontal gyrus co-varied with the lgi of the right superior temporal gyrus (STG) and the sulc of the inferior frontal gyrus. Additionally, alff of the bilateral STG and the right post-central gyrus co-varied with reho2 of the STG.

### Relevance to clinical research: a sub-study

**Fig. 5:**
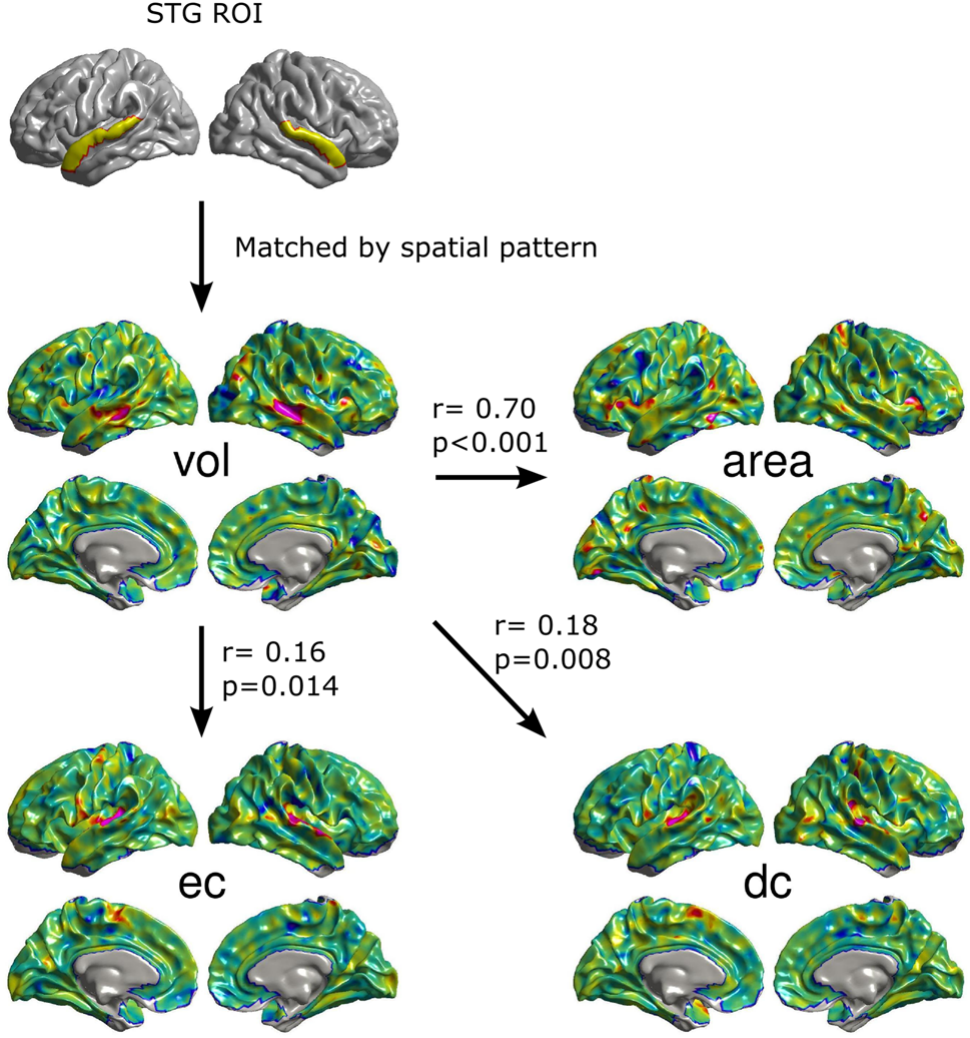
A sub-study focusing on the cortical volume of STG. Based on the commonly reported volume abnormality of STG in schizophrenia, we identified the vol SCM with the highest spatial consistency to the anatomical parcellation labeled ‘G_temp_sup-Lateral’ in FreeSurfer. The identified vol SCM (a member of MMCU6) predicts that accompanying deficits in the vol of STG, schizophrenia patients may have deficits in the area of bilateral inferior frontal gyri and the precuneus (*r* = 0.70) and deficits in the dc (*r* = 0.18) and ec (*r* = 0.16) of the STG

To demonstrate the potential contributions of the MMCUs to clinical research, we conducted a sub-study focusing on the gray matter volume of the STG (see Fig. 5). Abnormality in this structure has been robustly detected in schizophrenia patients (Shenton et al. 2001). We first identified the vol SCM with the highest spatial consistency with the anatomical parcellation labeled ‘*G_temp_sup*-*Lateral*’ in FreeSurfer. The identified vol SCM was a member of MMCU6 (Fig. S7). This MMCU indicated that the area, dc, and ec SCMs significantly co-varied with the vol SCM (Fig. 5). The correlation coefficients for these three SCMs were: area, *r* = 0.70 (*p* < 0.001), dc, *r* = 0.18 (*p* = 0.008), and ec, *r* = 0.16 (*p* = 0.014), suggesting that deficits of the area metric in bilateral inferior frontal gyri and the precuneus could be observable in schizophrenia patients and that deficits of dc and ec metrics in the STG may also occur in schizophrenia patients. Such an approach could be applied in studies of various mental disorders to detect potential deficits in the brain.

## Discussion

Although many structural and functional metrics of the brain have been developed and applied to a wide range of studies, the relationships across them have not been explicitly and systematically investigated. In this study, we filled this gap by mining two independent, large samples of healthy subjects. Applying gRAICAR to the first dataset (*N* = 184), we first proposed a set of hypotheses regarding the cross-subject co-variance among the 12 brain metrics, as represented by the MMCUs. We then found 42 of these MMCUs were reproducible in a second dataset (*N* = 340), and summarized them using an interactive database, where users could select any location on any metric to review the co-variance between the 12 metric maps. Using this database, we characterized the six functional metrics according to their general dependency to the six structural metrics. Further, we found relevance between some MMCUs and sex and handedness, suggesting that the MMCUs are meaningful in the neuroscience context and that the MMCUs are capable to link existing findings using different metrics. Moreover, based on a priori knowledge of abnormality in gray matter volume, the database predicted abnormalities in multiple functional metrics in schizophrenia. The implications and potential contributions of these findings are discussed below.

### A systematic view of structural–functional co-variance

This study provides a systematic view of the associations between multiple structural and functional metrics in healthy human brain, and advances our knowledge in three folds. First, the full picture of the relationships between six functional and six structural metrics brings in a novel avenue to characterize the functional metrics. A fundamental question for the neuroimaging field is what the functional metrics really tell us or what is their generative mechanism from the brain structure (Betzel et al. 2016). Many researchers have attempted to associate functional metrics to various phenotypes (Medaglia et al. 2015), but have not achieved a fully clear understanding of the biological meaning of these functional metrics. Our study offers a novel perspective that characterizes functional metrics based on their shared information with the structural metrics across individuals. Specifically, our results as in Fig. 2 suggest that alff weakly reflect variability in vol and area, dc has specific associations with vol, and ec has strong and specific associations with vol and area. The falff seems to carry unique information because it does not associate with any structural metrics. Surface-based reho reflects a combination of multiple structural metrics including area, sulc, curv, and thick. Reho2 is different from reho in that it has much weaker associations to structural metrics, echoing the nature of this local connectivity measure as a multi-modal, multi-scale neuroimaging marker of the human connectome (Jiang and Zuo 2015). Interestingly, since the reho metric reflects local coherence of the intrinsic neural activities (Zang et al. 2004), an insight from our finding is that only the coherence of activity within a close neighborhood (reho) reflects a synthesized variation of structural metrics, but not the coherence in a larger neighborhood (reho2). Another implication from Fig. 2 is that ec is the most structure-related functional metric, while falff is the most structure-irrelevant functional metric. Our systematic, unbiased investigation on large samples makes it possible to uncover these novel insights about the functional metrics.

Second, the present study extends the scope of structural–functional association investigations. Most studies linked structural and functional metrics based on their spatial correspondence (Liao et al. 2013; Seeley et al. 2009; Segall 2012; Zhang et al. 2011b; Vidal-Pineiro et al. 2014). The MMCUs found in our study suggest that the most reliably associated metric maps do not necessarily overlap in space. For instance, Fig. 3 indicates that multiple structural metrics (lgi, area, and vol) around the right SFG co-vary with the functional metrics (ec and alff). However, the SCMs of ec and alff showed different spatial distributions: while the ec SCM was consistent with the structural metrics, the alff SCM showed widespread contributions across the brain. This argument is supported by studies on mental disorders. For instance, gray matter abnormality in parietal and frontal regions in schizophrenia patients has been associated to the abnormal functional activations in bilateral temporal regions under an oddball task (Calhoun et al. 2006); abnormal correlation between the mean cortical thickness and the mean fractional anisotropy of the white matter has also been reported in schizophrenia patients (Sasamoto et al. 2014). Our study thus suggests a wider scope in searching for multi-modal imaging features, and provides evidence that the co-variance between structural and functional metrics may serve as an important factor for generative models of the human brain connectomes.

Third, our results provide the most detailed reference for selecting effective features for multi-modal neuroimaging applications. Nearly all of previous multi-modal imaging studies included no more than 3 imaging metrics (for a comprehensive review, see Sui et al. 2014). Our study suggests that each of the 12 frequently used metrics, especially the six functional metrics, carries some unique information (Fig. 2). Given the general principle that combining more unique information helps to improve the predictive power, our result that the functional metrics carry different information from the structural metrics suggests that combining more functional metrics may improve the performance of a machine-learning application. On the other hand, including more features in machine-learning models can lead to very high dimensionality and over-fitting issues. The reliable MMCUs in our study provide highly detailed reference for selecting effective features, and thus help to avoid the over-fitting problem. As an example, Fig. 4 indicates that the vol and area metrics in the middle frontal gyrus strongly co-vary with the lgi of the right posterior insula and the sulc of the inferior frontal gyrus, suggesting these four features are mutually dependent and three of them could be excluded in a machine-learning study. In other words, these MMCUs could constrain the search space for effective brain imaging features in machine-learning applications.

### Integrating/predicting findings from different metrics

The co-variance of structural metrics has previously been investigated in a number of studies (for a comprehensive review, see Alexander-Bloch et al. 2013a), in which the co-variance between the morphology of remote brain regions was examined across subjects. Based on these studies, structural co-variance has been associated with white matter tracts (Colibazzi et al. 2008; Gong et al. 2012; Lerch 2006), rates of cortical development (Alexander-Bloch et al. 2013b; Raz et al. 2005; Raznahan et al. 2011), genetic factors (Rimol et al. 2010; Schmitt et al. 2008; Thompson et al. 2001; Wright et al. 2002), functional networks (Alexander-Bloch et al. 2013b), and behaviors, such as intelligence (Lerch 2006) and musical ability (Bermudez et al. 2009; Jancke et al. 2012; Lv 2008). These findings indicate that the structural co-variance is a promising way for understanding the human brain organization.

Our results extend the existing structural co-variance studies towards multi-modal, multi-metric co-variance studies: in addition to structural metrics, we considered a variety of functional metrics to quantitatively and simultaneously examine the cross-subject co-variance of structural and functional properties. Given that functional metrics co-varying (but not necessarily overlapping) with structural metrics are more likely to be a trait marker that is robust against the state at the time of scan (Kelly et al. 2012; Castellanos et al. 2013; Zuo and Xing 2014), an important contribution of the current study is that we could find a number of detailed functional metric maps as candidates for individual trait markers.

The connected 12 metrics in each MMCU could serve as a reference to link existing findings using different metrics and to predict features of unexamined metrics. In this study, we showed an MMCU representing individual variability in sex and handedness (Fig. 4). This MMCU connects the vol, area, lgi, sulc, and reho2 metrics at different locations across the cortex. Most of these findings are echoed by existing studies, which have reported significant sex difference in the gray matter volume of the middle frontal gyrus (Ruigrok et al. 2014) and in the local gyrification index (Mutlu et al. 2013), the alff (Dong et al. 2010), and the reho of STG (Dai et al. 2012). This MMCU also predicts the sex effect on the sulcal depth of the inferior frontal gyrus. In fact, a study has linked the sulcal morphology and volume of Broca’s area (in the inferior frontal gyrus) to sex and handedness, indirectly supporting our prediction (Powell et al. 2012).

In the same vein, the MMCUs are capable to generate a number of hypotheses for multi-metric studies on mental disorders, as demonstrated in our sub-study focusing on the vol metric of the STG (Fig. 5). Schizophrenia literature has consistently pointed to gray matter volume abnormality in the STG (Shenton et al. 2001). Incorporating this knowledge with our database, we identified an MMCU that associates the vol metric of the STG to area, dc, and ec metrics. These associations hypothesize that the deficits of the area metric in bilateral inferior frontal gyri and the precuneus, and deficits of the dc and ec metrics in the STG may exist in schizophrenia patients. In fact, functional connectivity deficits of the STG have been frequently highlighted by schizophrenia studies (Leroux et al. 2014). Comparing these multi-metric associations between schizophrenia and the norm MMCU may provide additional insights to understand brain mechanisms for schizophrenia. The above examples evident two advantages of the proposed MMCUs: (1) provide a reference for building relationships between results using different metrics and (2) generate new hypotheses for specific metrics that have not been examined.

### Potential biological interpretations of MMCUs

Potential biological interpretations for the inter-metric MMCUs can be borrowed from those for the structural co-variance. In general, these phenomena can be interpreted using dependencies on the neurodevelopmental process (Alexander-Bloch et al. 2013a). Alexander-Bloch et al. speculated that the possible mechanisms underlying the co-variance between two regions are: sharing a developmental precursor (Riska 1986), inductive signaling from one developing tissue to another (Jackson 2010), simultaneous exposure to signals from a third party (Klingenberg 2005, 2008; Mitteroecker et al. 2012), shared genetic influences (Wright et al. 2002), correlational selection due to adaptive fitness (Roff and Fairbairn 2012; Sinervo and Svensson 2002), associations with the same behaviors (Kingsolver and Huey 2003), inherited ancestral relationships between phenotypic traits (Kirschner and Gerhart 1998), and common influences through environmental factors (Cheverud 1988; Waitt and Levin 1998). Compared with structural co-variance studies, the present study introduces an additional dimension, different structural/functional metrics, for consideration in the interpretation. Knowledge that bridges genetic factors, neural activities, and neuroimaging properties (Richiardi et al. 2015) might help investigations of the detailed mechanisms for these observations, serving as important topics for future studies (e.g., genetic basis and computational model of these MMCUs).

### Methodological issues

As argued by several studies, preprocessing pipelines (e.g., how to correct for motion artifacts) influence the results of functional MRI studies (Strother et al. 2004; Power et al. 2014; Aurich et al. 2015; Glatard et al. 2015). Consequently, differences in preprocessing pipelines should be taken into account when comparing other findings to our results (i.e., using our results as a reference for common structural–functional associations). To alleviate this issue, we chose a preprocessing strategy that was shown to generate graph-theoretical metrics with the maximal inter-subject reliability and minimal dependency on subject head motion among several commonly used preprocessing pipelines (Aurich et al. 2015). We argue that while different preprocessing pipelines have different considerations and advantages, they share an identical ultimate goal to produce results that are robust against confounding factors such as head motion, and that our choice of preprocessing pipeline is a maximal effort to reduce the influence of processing pipelines. As an additional effort to produce reproducible and useful results (Pernet and Poline 2015), we shared all datasets (FCP-Cambridge and CoRR-SWU), preprocessing pipelines such as CCS, gRAICAR,^3^ and other scripts^4^ to the public for researchers to tune the processing parameters at will in examining pipeline-specific/independent structural–functional associations.

Another important issue is that the dimensionality estimations in the ICA decomposition may impact the resultant MMCUs. To evaluate the impact, we repeated the discovery analysis with a range of fixed numbers of components in ICA, changing from 15 to 100 with an interval of 5. We calculated the similarity (correlation coefficients) between the resultant MMCUs and those reported above. The results showed that the similarity of MMCUs obtained with the changing dimensionality parameters did not change dramatically (shown in Supplementary Figure S17). Therefore, we believe the reported results are robust against the dimensionality parameter.

In summary, using a completely data-driven approach, we examined the association across six structural and six functional metrics according to the co-variance observed across subjects and presented an overview of the associations among multiple structural and functional metrics. The results could serve as a normative reference for multi-metric investigations, help to explain anatomical basis of the functional metrics, and provide insights for integrating multi-modal imaging markers for clinics.

## Electronic supplementary material

Below is the link to the electronic supplementary material.

Supplementary material 1 (PDF 10867 kb)

## Footnotes

1 The gRAICAR package is freely available at https://github.com/yangzhi-psy/gRAICAR. It is also a part of CCS (https://github.com/zuoxinian/CCS).

2 Also available at http://ddl.escience.cn/f/qMsE.

3 Available at https://github.com/yangzhi-psy/gRAICAR.

4 Available at https://github.com/yangzhi-psy/covGraph.
