# Supplementary material for: Brain structure–function associations identified in large-scale neuroimaging data
Source: Brain Struct Funct. 2016 Jan 9;221(9):4459–74. doi: 10.1007/s00429-015-1177-6 (PMC5102954; doi:10.1007/s00429-015-1177-6)
Supplement: Supplementary file 1 — Supplementary material 1 (PDF 10867 kb) [file 429_2015_1177_MOESM1_ESM.pdf]

Supplementary Materials

Brain structure-function associations identified in large-scale neuroimaging data

Zhi Yang*, Jiang Qiu, Peipei Wang, Rui Liu, Xi-Nian Zuo*

Corresponding authors:

Zhi Yang (yangz@psych.ac.cn); Xi-Nian Zuo (zuoxn@psych.ac.cn)

Address: 16 Lincui Road, Chaoyang District, Beijing, 100101, China

Content

Supplementary Text 1: Abbreviations 1

Supplementary Text 2: Details of Functional Metrics 3

Supplementary Text 3: Details of Structural Metrics 7

References 9

Supplementary Table 1 11

Supplementary Figures 1-17 12

Supplementary Text 1: Abbreviations

1. Structural metrics

Abbreviation	Full Spelling
vol	Cortical volume
area	Surface area
thick	Cortical thickness
curv	Mean curvature
lgi	Local gyrification index
sulc	Sulcal depth

2. Functional metrics

Abbreviation	Full Spelling
alff	Amplitude of low frequency fluctuation
falff	Fractional amplitude of low frequency fluctuation
dc	Degree centrality
ec	Eigenvector centrality
reho	Regional homogeneity
reho2	Regional homogeneity with extended scope

3. Datasets and scan parameters

Abbreviation	Full Spelling
FCP	1000 Functional Connectomes Project
INDI	International Neuroimaging Data-Sharing Initiative
FCP-Cambridge	Cambridge-Buckner site in FCP
CoRR	Consortium for Reliability and Reproducibility
CoRR-SWU	Southwest University (China) site in CoRR
MP-RAGE	3D Magnetization Prepared Rapid Gradient Echo
rfMRI	resting-state functional MRI
EPI	echo-planar imaging
FOV	field of view

4. General data processing terms

Abbreviation	Full Spelling
CCS	Connectome Computation System
ICA	Independent Component Analysis
CSF	cerebrospinal fluid
GM	gray matter
WM	white matter
FWHM	full width half magnitude
BBR	boundary-based registration
maxTran	the maximum distance of translational movement
maxRot	the maximum degree of rotational movement
meanFD	the mean frame-wise displacement
mcBBR	the minimal cost of the BBR co-registration

5. gRAICAR related terms

Abbreviation	Full Spelling
gRAICAR	Generalized Ranking and Averaging Independent Component Analysis
SCM	Surface Component Map
FSM	Full Similarity Matrix
MMCU	Multi-Metric Covariance Unit
ICC	Intra-class Correlation Coefficient

Supplementary Text 2: Details of Functional Metrics

In this study, we employed six metrics to characterize functional networks (for more details of their definitions, see our recent review in functional connectomics from Zuo and Xing, 2014). All of these metrics are defined on each vertex of the cortical surface. The alff and falff reflect local fluctuations on each vertex, the dc and ec reveals the role of each vertex in the entire functional network, and the reho and reho2 metrics describe local functional coherence of the network. Details on definitions, computations, and biological meanings for these metrics are summarized below:

1. ALFF

Amplitude of low-frequency fluctuations (ALFF) characterizes the amplitude of fluctuations of voxel-wise low-frequency signal in resting-state fMRI (rfMRI) time series (Zang et al., 2007). It indicates the strength or intensity of the low-frequency fluctuations by summing up the total root squared power within the frequency range between 0.01 and 0.1Hz. This metric is test-retest reliable (Zuo et al., 2010) and has been assigned with the biological meaning of the moment-to-moment variability of human brain function (Garrett et al., 2013, 2010) as well as the associations with task-induced brain activity and behavioral performance (Mennes et al., 2011; Zou et al., 2013).

In the current study, the ALFF maps were computed on cortical surface vertex-wise. First, the individual preprocessed 4D rfMRI time series were projected onto the *fsaverage* standard cortical surface with 163,842 vertices per hemisphere. The average distance between neighbor vertices was 1 mm. The data were then down-sampled onto the *fsaverage5* standard cortical surface (average distance between neighbor vertices: 3.8 mm), which contained 10,242 vertices per hemisphere (Yeo et al. 2011). Fast Fourier transform were performed on the time times for each vertex, and ALFF metrics were then computed by averaging the square root of the power spectrum at frequencies between 0.01-0.1Hz for all vertices.

2. fALFF

Fractional ALFF (fALFF) is a normalized version of ALFF, where ALFF measured within the low-frequency range (0.01-0.1Hz) is divided by the total power in the entire detectable frequency range (Zou et al., 2008). It reveals the relative contribution of low frequency fluctuations against the entire rfMRI time

series. As demonstrated by Zuo et al. (2010), fALFF is less affected by physiological noise when compared to ALFF. In the present study, the computation of fALFF was also based on vertex-wise time series. Following the procedures described in the ALFF section, we divided vertex-wise ALFF within 0.01-0.1Hz range by the ALFF derived from the whole frequency range to generate fALFF maps.

3. DC

The degree centrality (DC) is a graph-theoretic metric. The brain network is modeled as a weighted graph, where each vertex is a node and the functional coherence between pairs of vertices is defined as edge. Given a node, its DC is defined as the sum of weights from edges connecting to the node (also referred to as nodal strength). In our analysis, we used Pearson's Correlation to measure the functional coherence between time series from pairs of different vertices:

$$dc(i) = \sum_{\substack{j=1 \\ j \neq i}}^{N} r_{ij} \ ,$$

where i denotes the current vertex, and j indices all vertices besides i, and r_{ij} represents Pearson's correlation coefficient between time series on vertices i and j. The DC surface maps thus indicate the strength of full pictures of connections vertex-wise. This metric has been widely used to examine local property of brain networks (Fransson et al. 2011; Zuo et al., 2012).

4. EC

The eigenvector centrality (EC) is another graph-theoretical metric for brain networks proposed by Bonacich (1972). It is defined as the first eigenvector of the adjacency matrix:

$$ec(i) = \mu_1(i) = \frac{1}{\lambda_1} \sum_{j=1}^{N} r_{ij} \mu_1(j) \ ,$$

where λ_1 represents the first eigenvalue of the adjacency matrix, and r_{ij} indicates the Pearson's correlation coefficient between vertices i and j. This metric does not only depend on the strength of connection on the current node, but also influenced by the EC of the nodes connecting to the current node. This recursive nature allows taking the global features of the graph into

consideration in the local property. It metric has been increasingly used to characterize brain networks (Fagerholm et al., 2015; Wheeler et al., 2015). Same as the above metric, this metric was computed on cortical surface, where each node was defined as a vertex.

5. ReHo

The regional homogeneity (ReHo) is used to characterize local functional homogeneity that indicates boundaries between functional heterogeneous regions (Zang et al., 2004). The homogeneity of cell number/type and neuron density most likely contributes to functional homogeneity within a small region (Lichtman and Denk, 2011); thus, this regional variation in micro-level homogeneity could also contribute to the regional variation in local functional homogeneity (Jiang et al., 2015). The ReHo metric was initially computed in whole-brain 3D volume space by calculating Kendall’s coefficients of concordance between time series on neighbor voxels of a center voxel.

In this study, we computed surface-based reho (Zuo et al., 2013), where for each vertex, the time series of its nearest 6 neighbors (directly connected in topology, length-one neighbors) were used to compute the Kendall’s coefficient of concordance:

$$\mathrm{reho} = \frac{\sum_{i=1}^{n} R_i^2 - n(\bar{R})^2}{\frac{1}{12}K^2(n^3 - n)} ,$$

where R_i represents the ranks of the value in time series $v_i(t)$, n is the number of time points, $\bar{R}_i$ is the mean rank across its neighbors at the ith time point, and $\bar{R}$ is the overall mean rank across all neighbors and across all time points. This computation is repeated for every vertex that has BOLD time series to produce a vertex-wise local functional homogeneity surface map. The reho computation is intrinsically a type of spatial and temporal smoothing operation on the cortical surface that is helpful for suppressing the time series noise and for mitigating the inter-individual spatial normalization issue.

6. ReHo2

The meaning of ReHo2 is similar to ReHo. The only difference in ReHo2 is that the Kendall’s coefficients of concordance were computed using 19 neighbor

vertices of the central vertex, which further included the second layer of neighbor vertices (length-two neighbors) that directly connect to the first layer of 6 neighbor vertices used in ReHo metric above. This metric extends the scope in measuring local functional homogeneity and is more comparable to the classic volume-based ReHo that uses 26 neighbor voxels.

Supplementary Text 3: Details of Structural Metrics

We used six structural (morphological) metrics in this study, which were all computed by FreeSurfer. These metrics are well established, widely used, and easily computed with standard software. Here we list the definitions of these metrics. These definitions are from Rettmann et al. (2006) and the FreeSurfer Wiki:

1. AREA

The area metric on each vertex indicates the average area of all the triangles the vertex is associated with. A larger value of this metric suggests that the current vertex represents (occupies) relatively larger area on the cortex.

2. THICK

The thickness (THICK) metric reflects the distance between the grey-white matter boundary mesh and the pial mesh corresponding to the given vertex. Computationally, at each vertex, the nearest point on each surface to the other (grey-white matter boundary mesh and pial mesh) is identified, and the THICK metric is the average distance between the nearest points.

3. VOL

The volume (VOL) metric is the product of THICK and AREA metrics on the given vertex.

4. CURV

The mean curvature (CURV) metric on each vertex is defined as the mean of the maximum and minimum principle curvatures observed around the given vertex. A positive value of the CURV metric indicates the surface is bending outward, and a negative CURV indicates an inward bending.

5. LGI

The local gyrification index (LGI) is used to quantify the amount of cortex buried within the sulcal folds as compared with the amount of cortex on the outer visible cortex. A cortex with extensive folding has a large gyrification index, whereas a cortex with limited folding has a small gyrification index. LGI is computed by dividing the area of the pial surface by the area of the outer

smoothed surface corresponding to the same vertex.

6. SULC

The sulcal depth (SULC) metric on each vertex is defined as the length of the shortest path, along the surface, from the given vertex to the outer smooth surface of the cortex.

References

Fagerholm, E.D., Hellyer, P.J., Scott, G., Leech, R., Sharp D.J., 2015. Disconnection of network hubs and cognitive impairment after traumatic brain injury. Brain 138, 1696-1709.

Fransson, P., Aden, U., Blennow, M., Lagercrantz, H., 2011. The functional architecture of the infant brain as revealed by resting-state fMRI. Cereb Cortex 21,145-154.

Garrett, D.D., Kovacevic, N., McIntosh, A.R., Grady, C.L., 2010. Blood oxygen level-dependent signal variability is more than just noise. J Neurosci 30, 4914-4921.

Garrett, D.D., Samanez-Larkin, G.R., MacDonald, S.W., Lindenberger, U., McIntosh, A.R., Grady, C.L., 2013. Moment-to-moment brain signal variability: a next frontier in human brain mapping? Neurosci Biobehav Rev 37, 610-624.

Jiang, L., Xu, T., He, Y., Hou, X.H., Wang, J., Cao, X.Y., Wei, G.X., Yang, Z., He, Y., Zuo, X.N., 2015. Toward neurobiological characterization of functional homogeneity in the human cortex: regional variation, morphological association and functional covariance network organization. Brain Struct Funct 220: 2485-2507.

Lichtman, J.W., Denk, W., 2011. The big and the small: challenges of imaging the brain's circuits. Science 334, 618-623.

Mennes, M., Zuo, X.N., Kelly, C., Di Martino, A., Zang, Y.F., Biswal, B., Castellanos, F.X., Milham, M.P., 2011. Linking inter-individual differences in neural activation and behavior to intrinsic brain dynamics. Neuroimage 54, 2950-2959.

Rettmann, M.E., Kraut, M.A., Prince, J.L., Resnick, S.M., 2006. Cross-sectional and longitudinal analyses of anatomical sulcal changes associated with aging. Cereb Cortex 16, 1584-1594.

Wheeler, A.L., Wessa, M., Szeszko, P.R., Foussias, G., Chakravarty, M.M., Lerch, J.P., DeRosse, P., Remington, G., Mulsant, B.H., Linke, J., Malhotra, A.K., Voineskos, A.N., 2015. Further neuroimaging evidence for the deficit subtype of schizophrenia: a cortical connectomics analysis. JAMA Psychiatry 72, 446-455.

Yeo, B.T., Krienen, F.M., Sepulcre, J., Sabuncu, M.R., Lashkari, D.,

Hollinshead, M., Roffman, J.L., Smoller, J.W., Zöllei, L., Polimeni, J.R., Fischl, B., Liu, H., Buckner, R.L., 2011. The organization of the human cerebral cortex estimated by intrinsic functional connectivity. J Neurophysiol 106, 1125-1165.

Zang, Y.F., He, Y., Zhu, C.Z., Cao, Q.J., Sui, M.Q., Liang, M., Tian, L.X., Jiang, T.Z., Wang, Y.F., 2007. Altered baseline brain activity in children with adhd revealed by resting-state functional MRI. Brain Dev 29, 83-91.

Zang, Y., Jiang, T., Lu, Y., He, Y., Tian, L., 2004. Regional homogeneity approach to fMRI data analysis. Neuroimage 22, 394-400.

Zou, Q., Ross, T.J., Gu, H., Geng, X., Zuo, X.N., Hong, L.E., Gao, J.H., Stein, E.A., Zang, Y.F., Yang, Y., 2013. Intrinsic resting-state activity predicts working memory brain activation and behavioral performance. Hum Brain Mapp 34, 3204-3215.

Zou, Q., Zhu, C.Z., Yang, Y, Zuo, X.N., Long, X.Y., Cao, Q.J., Wang, Y.F., Zang, Y.F., 2008. An improved approach to detection of amplitude of low-frequency fluctuation (ALFF) for resting-state fMRI: fractional ALFF. J Neurosci Methods 172, 137-141.

Zuo, X.N., Ehmke, R., Mennes, M., Imperati, D., Castellanos, F.X., Sporns, O., Milham, M.P., 2012. Network centrality in the human functional connectome. Cereb Cortex 22, 1862-1875.

Zuo, X.N., Kelly, C., Adelstein, J.S., Klein, D.F., Castellanos, F.X., Milham, M.P., 2010. Reliable intrinsic connectivity networks: Test-retest evaluation using ICA and dual regression approach. Neuroimage 49, 2163-2177.

Zuo, X.N., Xing, X.X., 2014. Test-retest reliabilities of resting-state fMRI measurements in human brain functional connectomics: A systems neuroscience perspective. Neurosci Biobehav Rev 45, 100-118.

Zuo, X.N., Xu, T., Jiang, L., Yang, Z., Cao, X.Y., He, Y., Zang, Y.F., Castellanos, F.X., Milham, M.P., 2013. Toward reliable characterization of functional homogeneity in the human brain: Preprocessing, scan duration, imaging resolution and computational space. Neuroimage 65, 374-386.

Supplementary Table

Table S1. Number of independent components for 12 structural and functional metrics

Structural Metrics						Functional Metrics					
vol	area	curv	thick	sulc	lgi	alff	dc	ec	reho	reho2	falff
67	65	26	12	99	88	85	80	11	41	27	27

Supplementary Figures

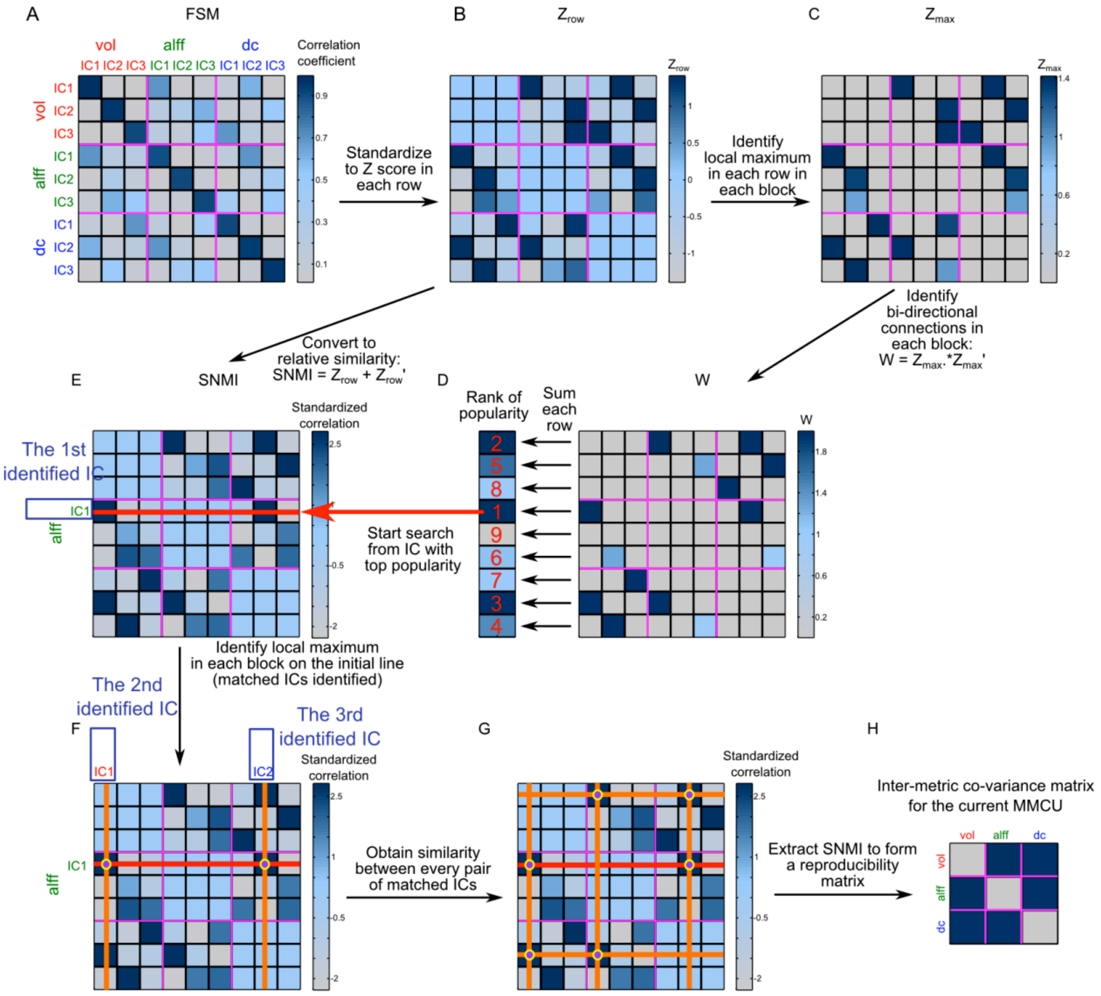

Figure S1. Demonstration of gRAICAR algorithm used to propose multi-metric co-variance units (MMCUs). Here we use surface component maps (SCMs) from three different metrics, vol, alff, and dc as example to demonstrate the workflow. Assuming there are three SCMs obtained in each metric, as indicated by "IC1", "IC2", and "IC3". (A) gRAICAR first constructed a full similarity matrix (FSM) containing pair-wise similarity between all the SCMs from the 3 metrics. The similarity between the SCMs is defined as the Pearson's correlation coefficients between their subject courses. The FSM is segmented into metric-blocks by purple lines, so that the FSMs from the same metric stayed together and the similarity values between them were represented in a block within the FSM. (B) The similarity values are then converted into relative values (Zrow) within each row in each metric-block. (C) The maximal value in each row of each metric-block in Zrow is then retained in a new matrix (Zmax),

and all other values are set to 0. Each row in the Zmax matrix thus reveals the most similar SCMs from different metrics to the SCM represented in current row. (D) The Zmax matrix is multiplied to its transform (multiply elements in corresponding locations). This operation eliminates unpaired inter-metric maximal similarities. In the resultant matrix, the sum of each row yields the “popularity” value for each SCM, representing a special form of centrality that takes specificity and mutual correspondence of SCMs into account. (E) A standardized FSM is formed by adding the Zrow matrix to its transpose, so that the similarity between a pair of SCMs from different metrics was standardized among all SCMs from the relevant metrics. (F) Starting from the top SCM in the popularity rank, 3 SCMs from different metrics were identified in the standardized FSM, by searching for maxima in metric-blocks on the top SCM’s row. The blue texts depict where the SCMs are identified in the standardized FSM.

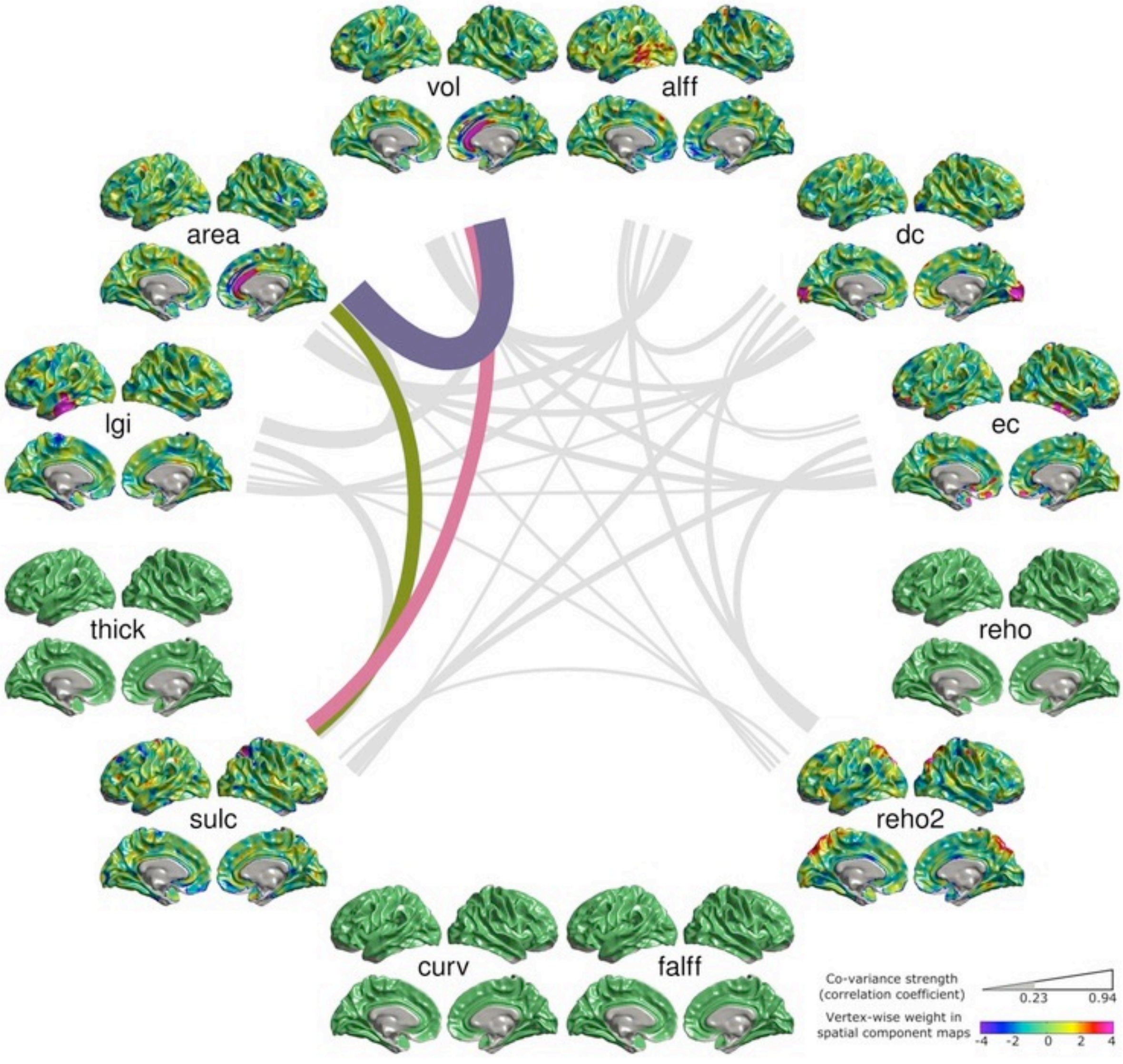

Figure S2. MMCU1, ICC = 0.89, CC = 0.93

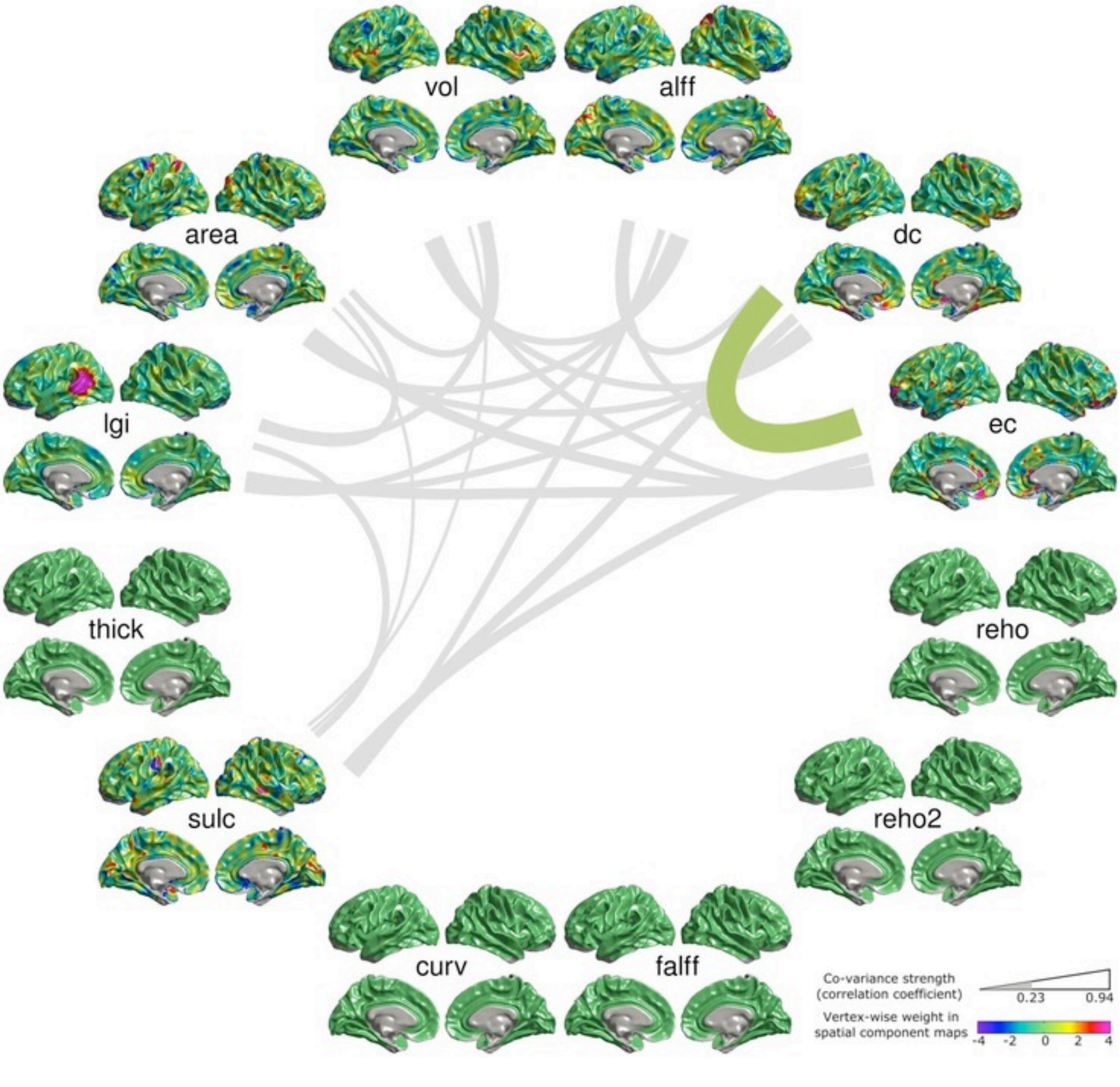

Figure S3. MMCU2, ICC = 0.81, CC = 0.90

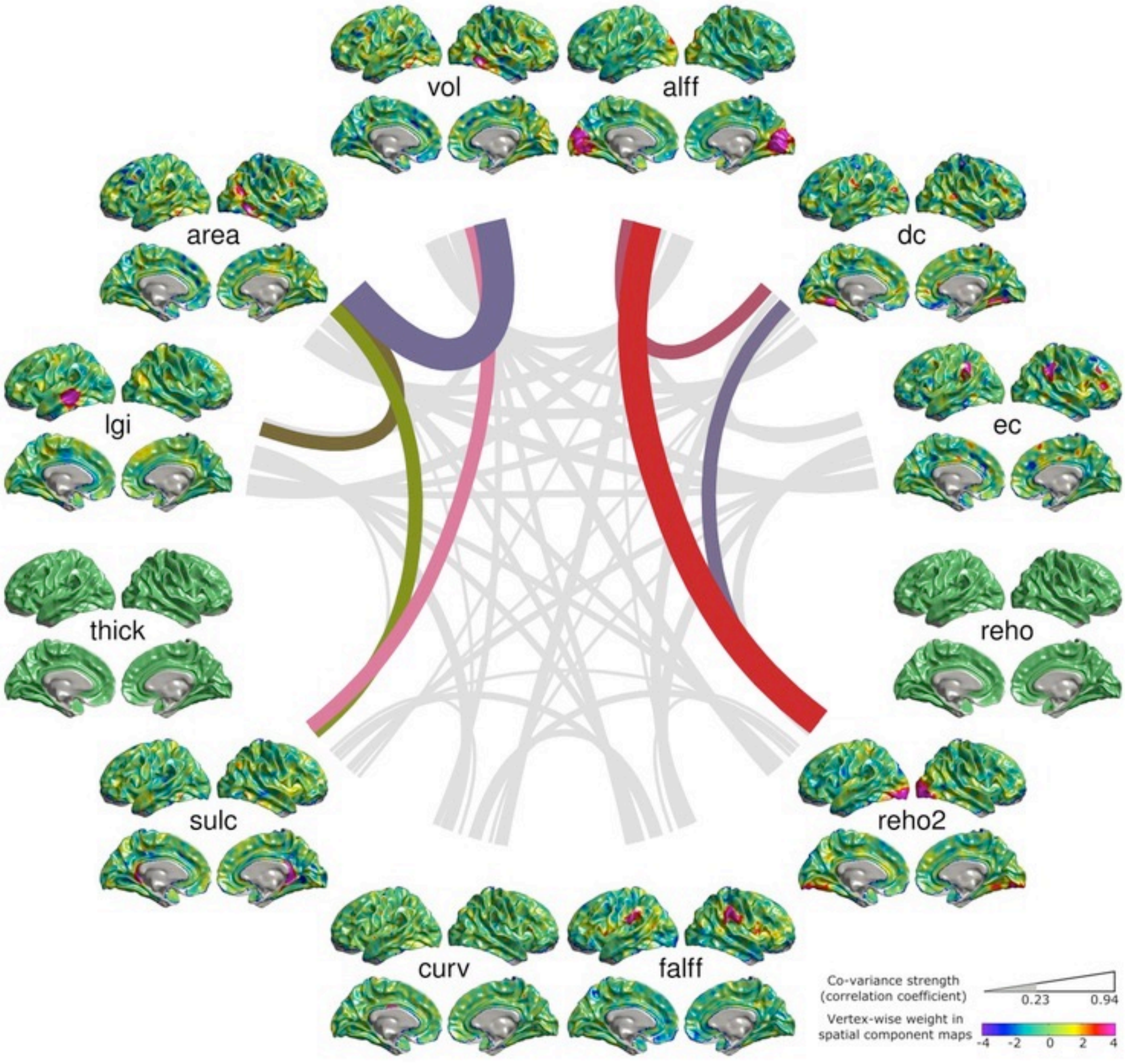

Figure S4. MMCU3, ICC = 0.87, CC = 0.89

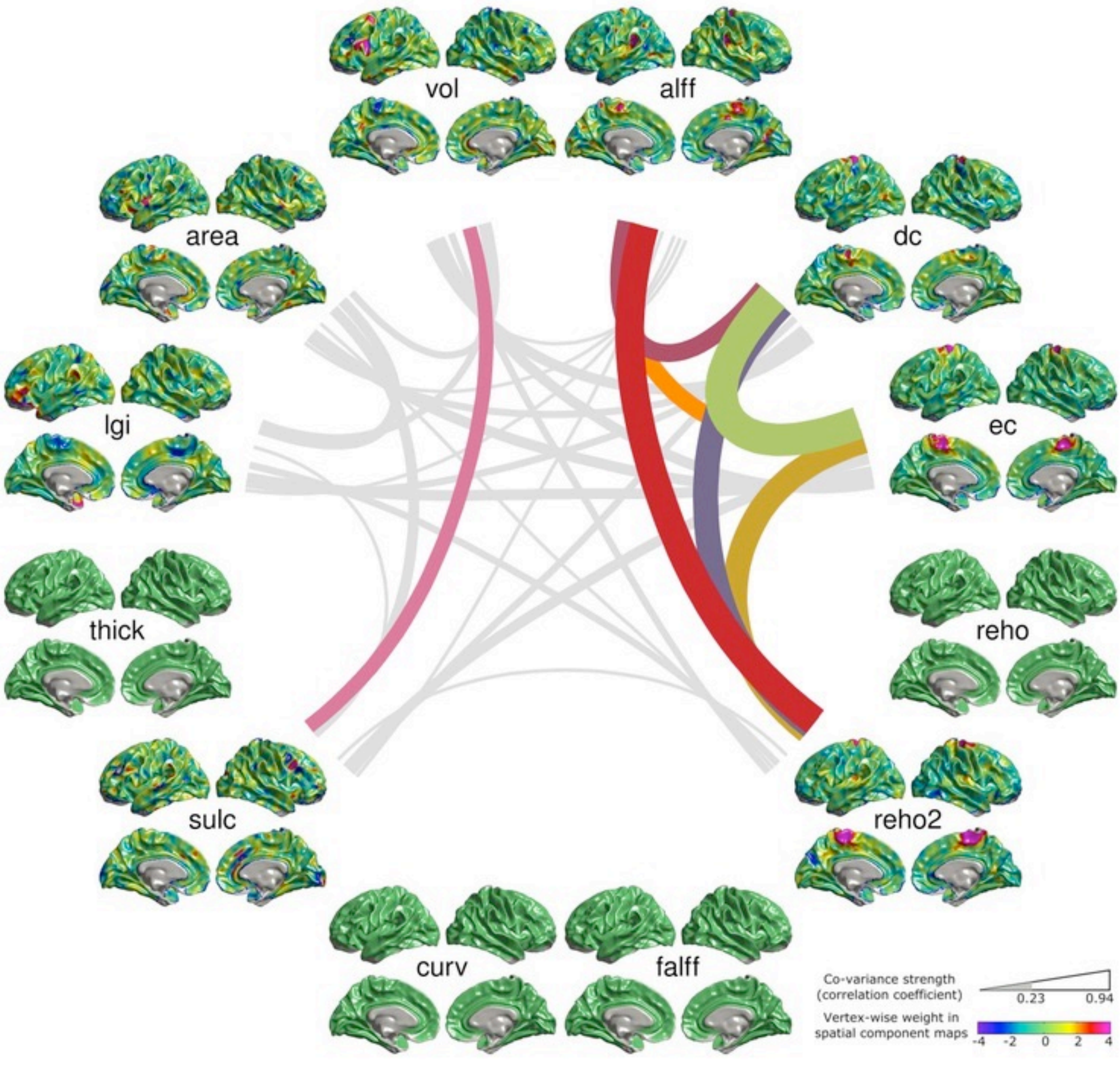

Figure S5. MMCU4, ICC = 0.82, CC = 0.87

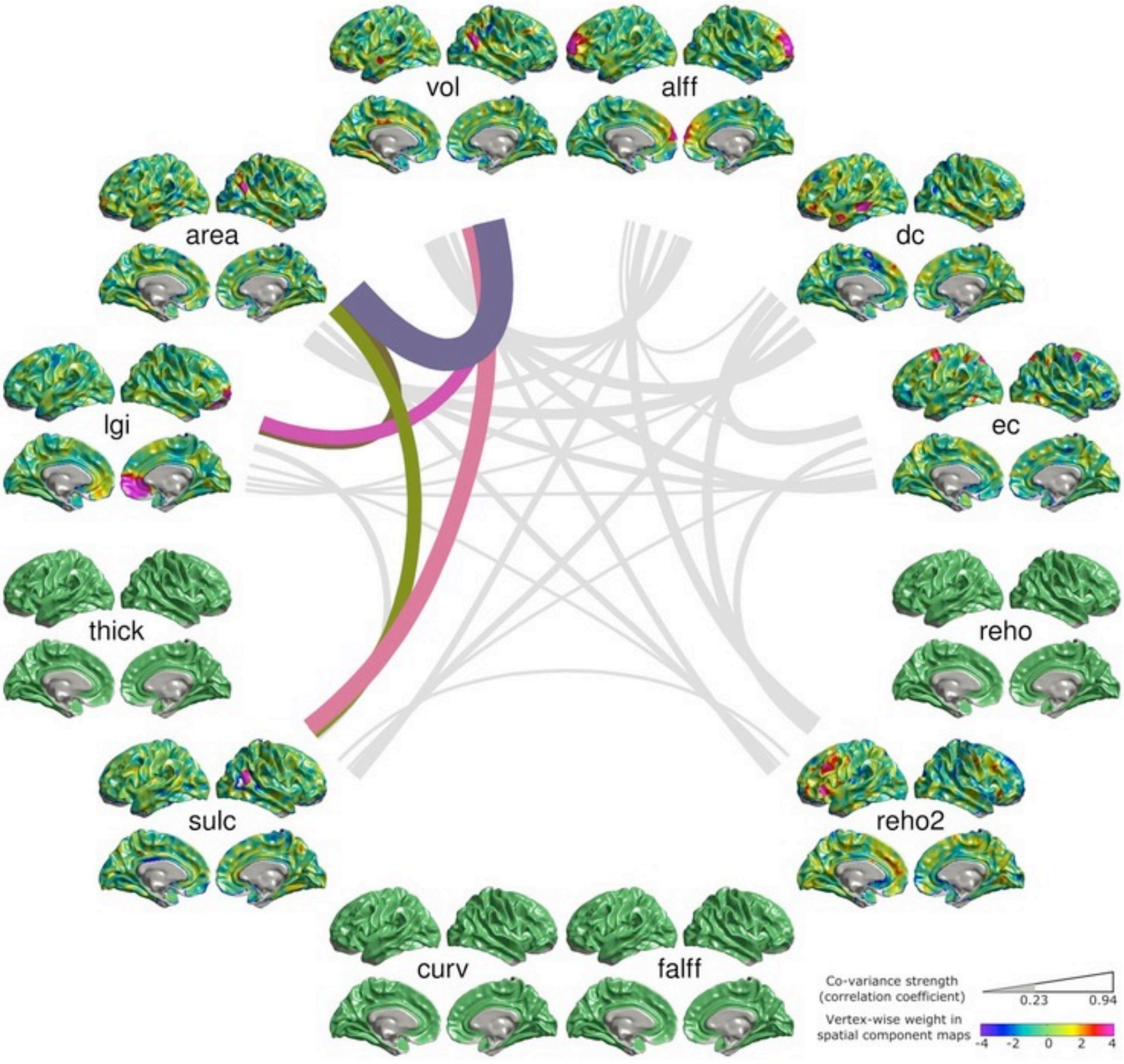

Figure S6. MMCU5, ICC = 0.85, CC = 0.88

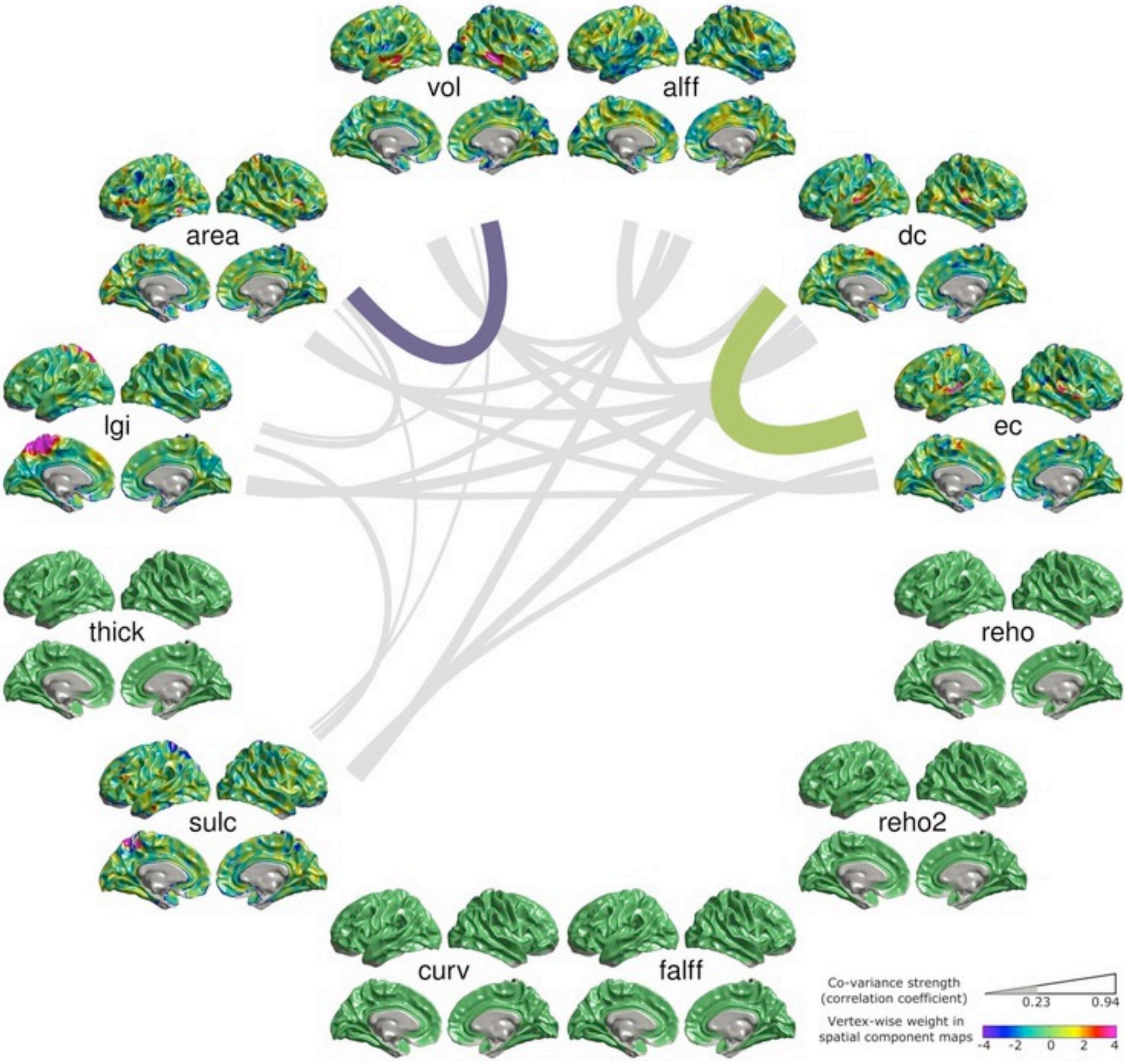

Figure S7. MMCU6, ICC = 0.86, CC = 0.88

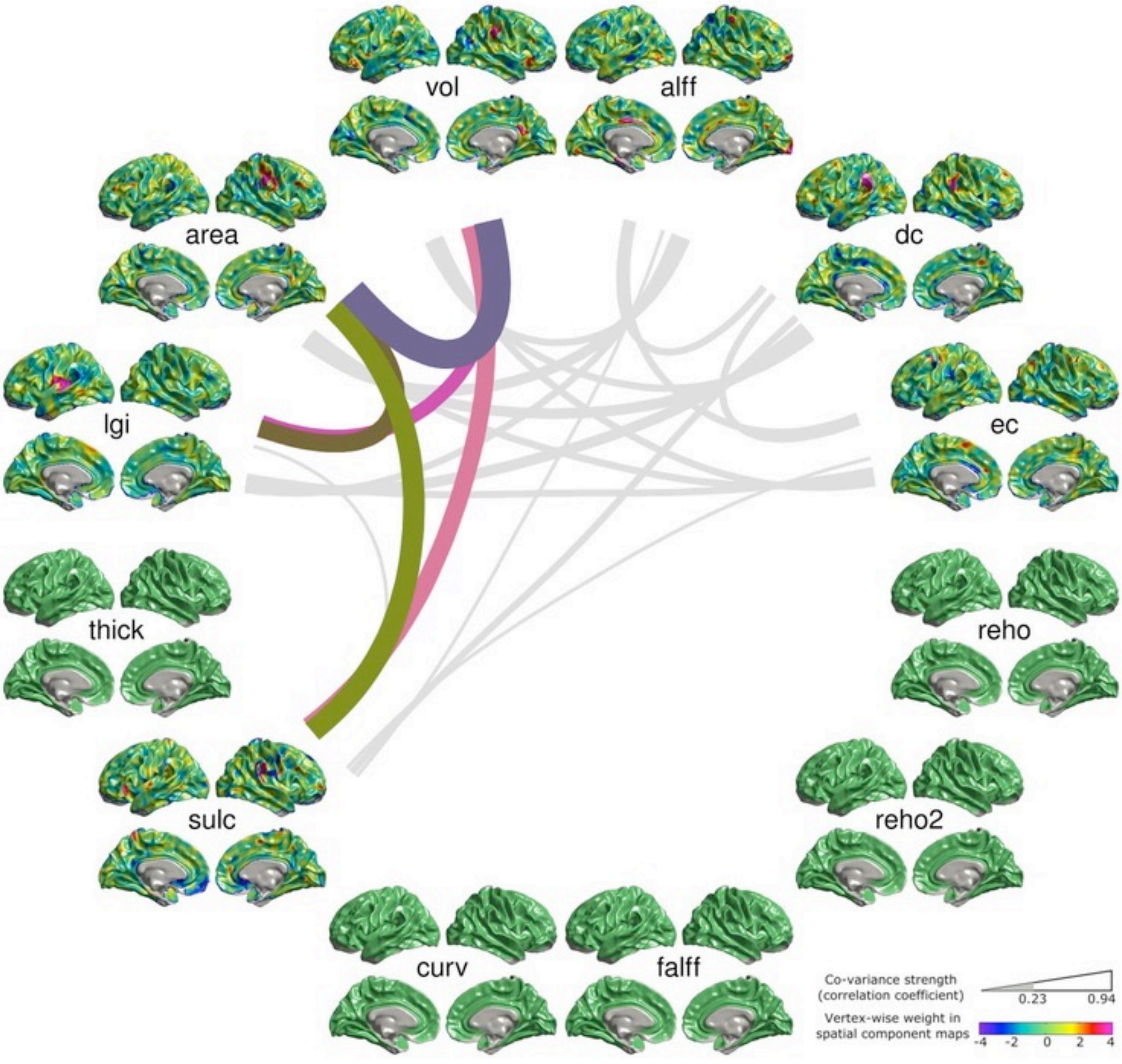

Figure S8. MMCU7, ICC = 0.85, CC = 0.88

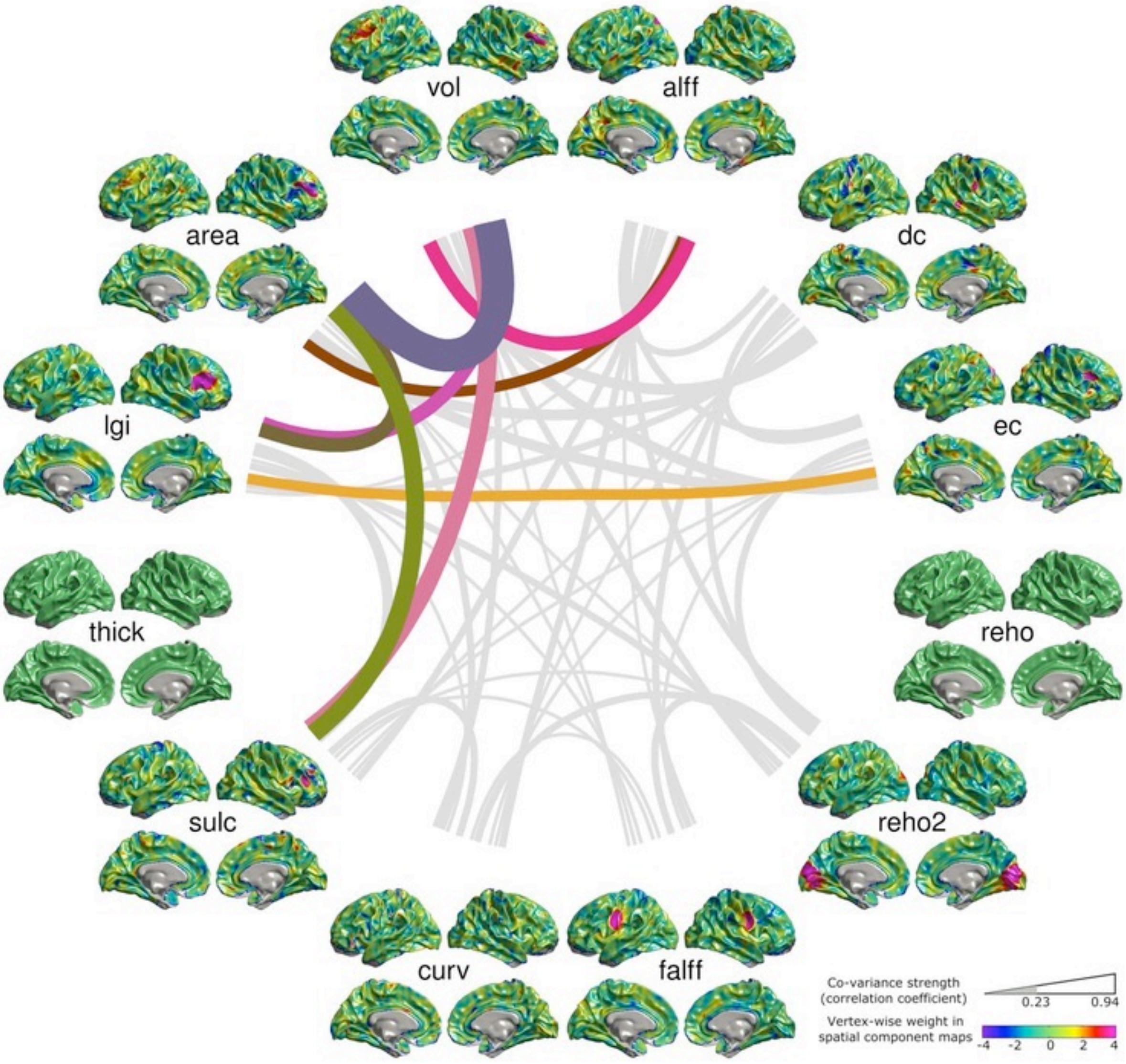

Figure S9. MMCU8, ICC = 0.87, CC = 0.88

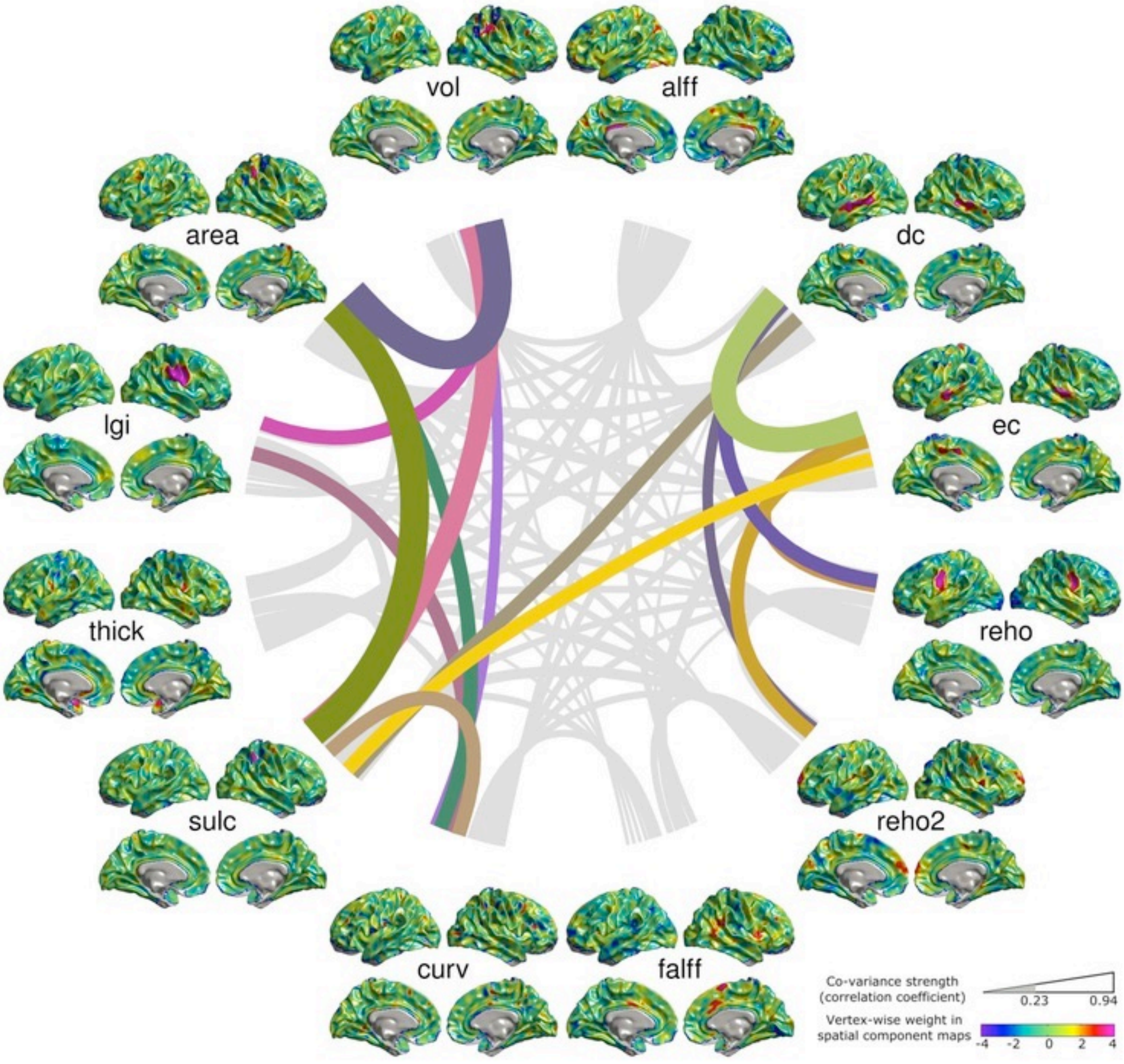

Figure S10. MMCU9, ICC = 0.83, CC = 0.86

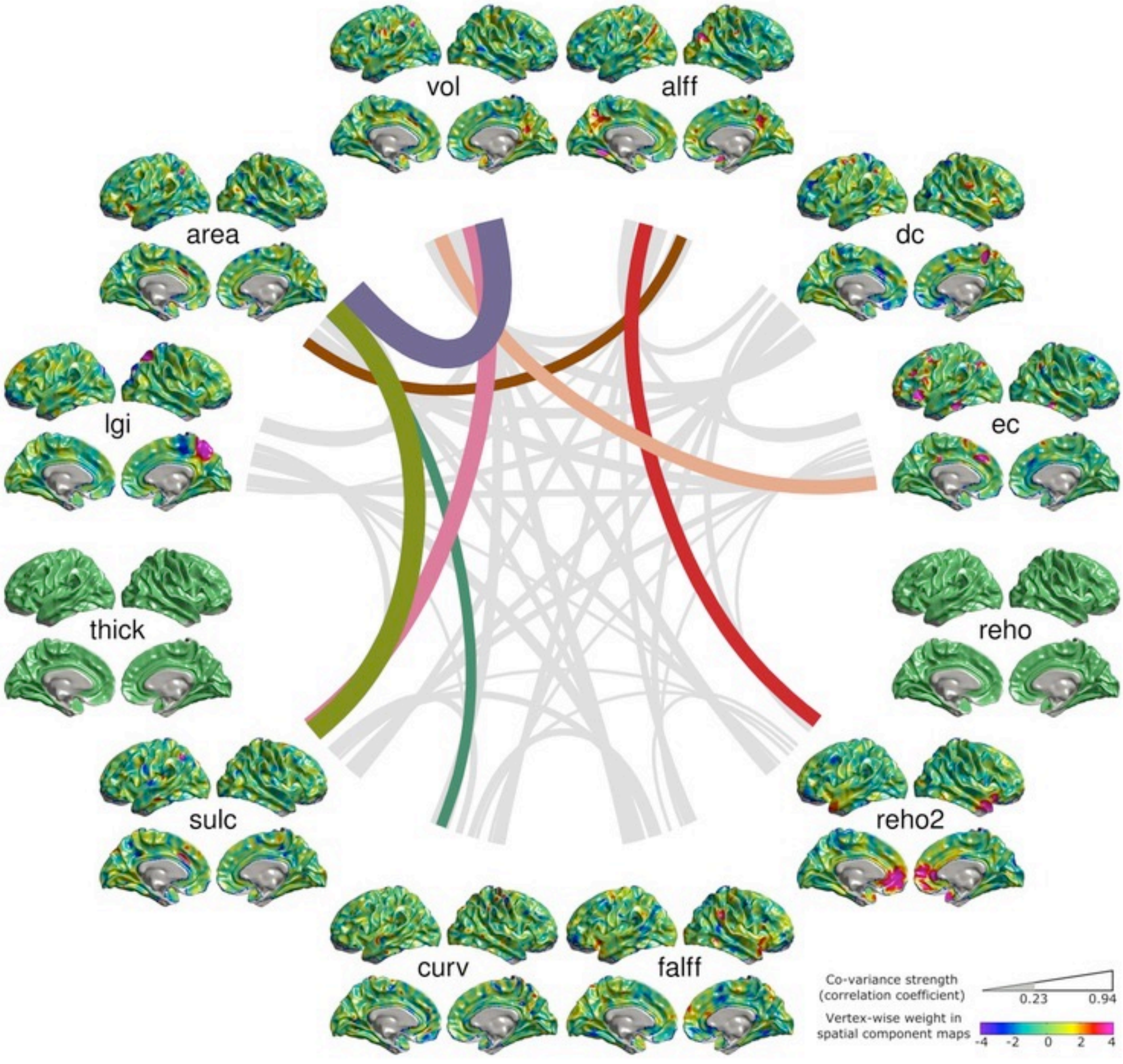

Figure S11. MMCU10, ICC = 0.82, CC = 0.85

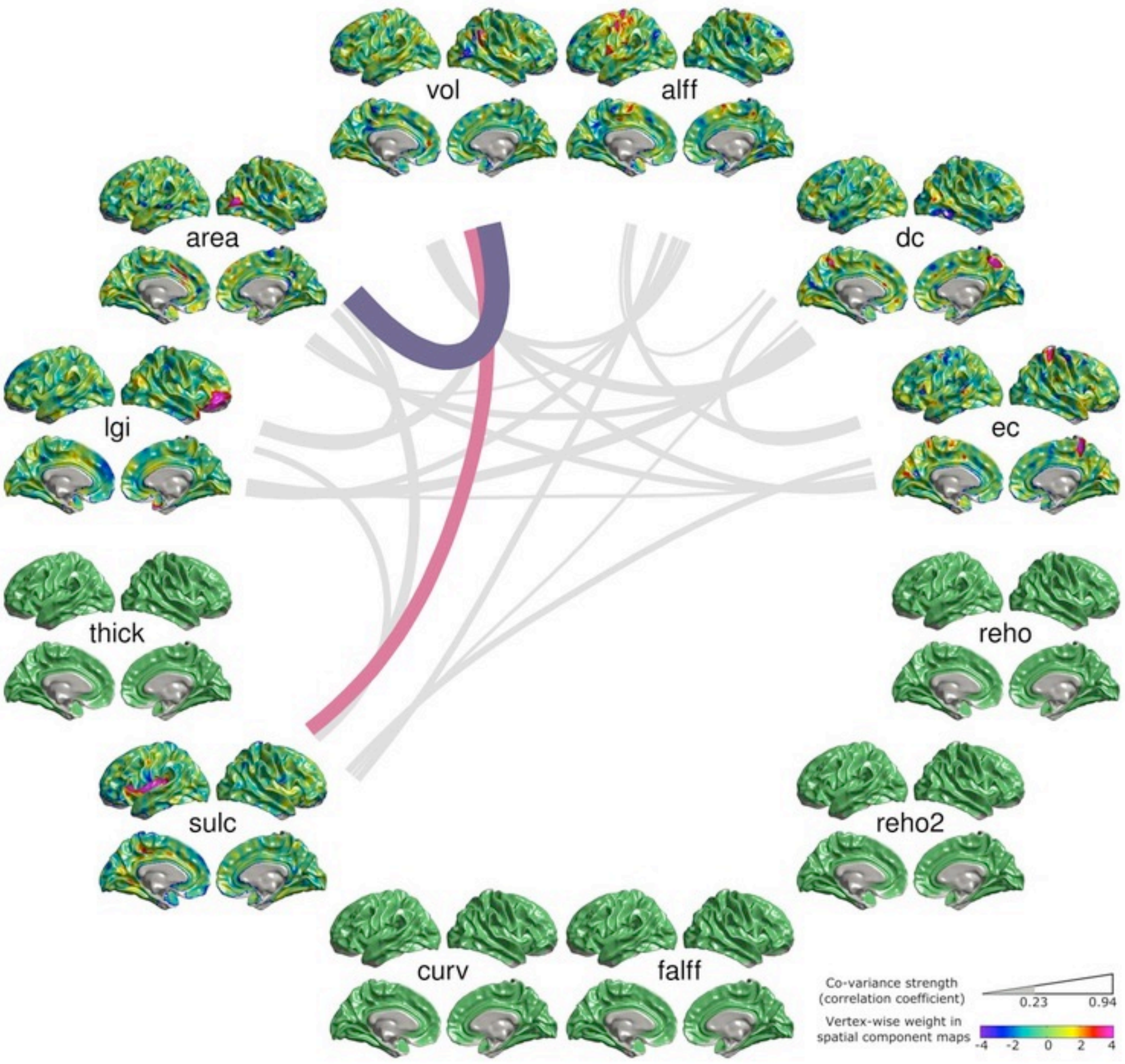

Figure S12. MMCU11, ICC = 0.81, CC = 0.82

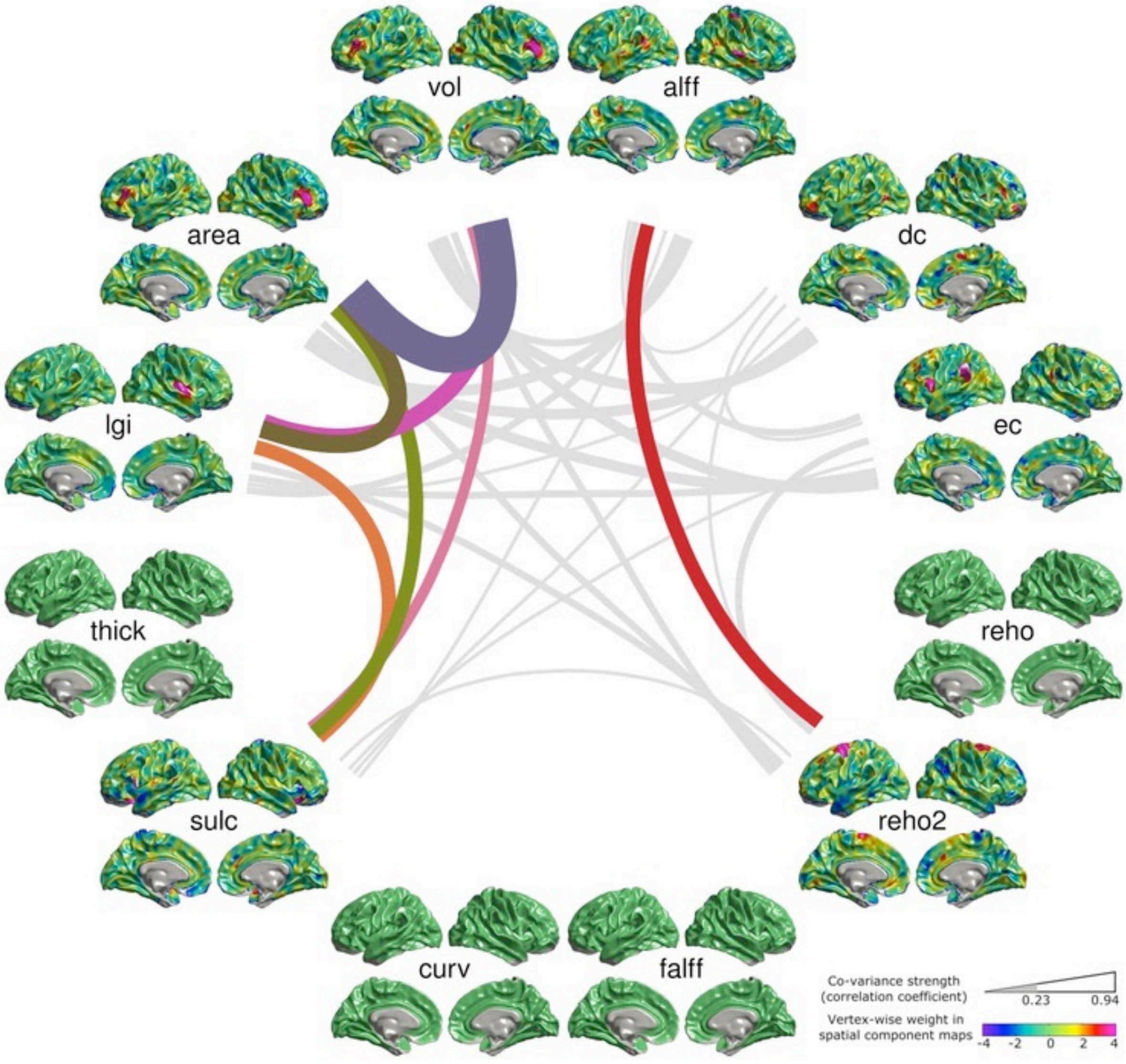

Figure S13. MMCU12, ICC = 0.87, CC = 0.89

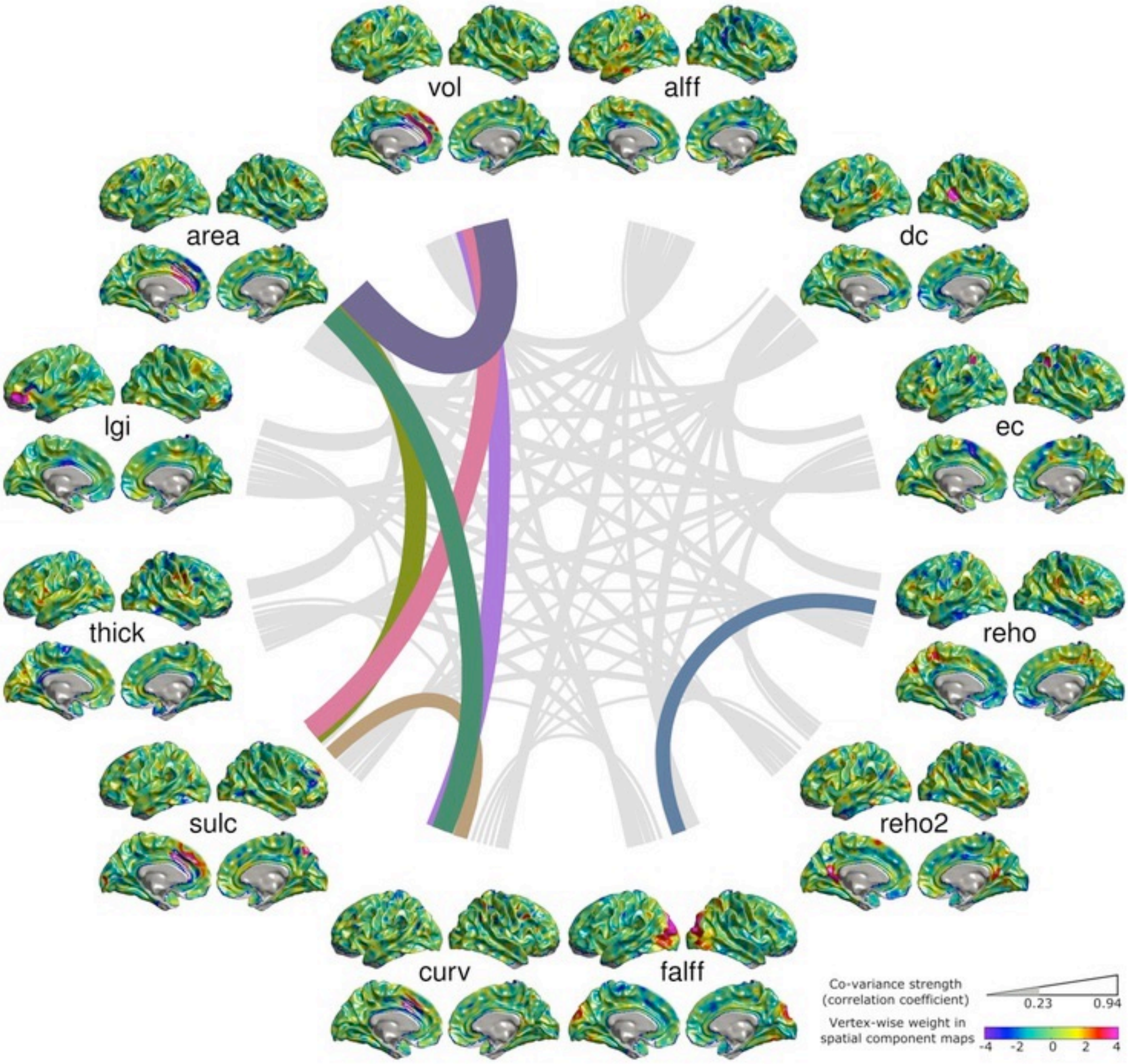

Figure S14. MMCU13, ICC = 0.83, CC = 0.83

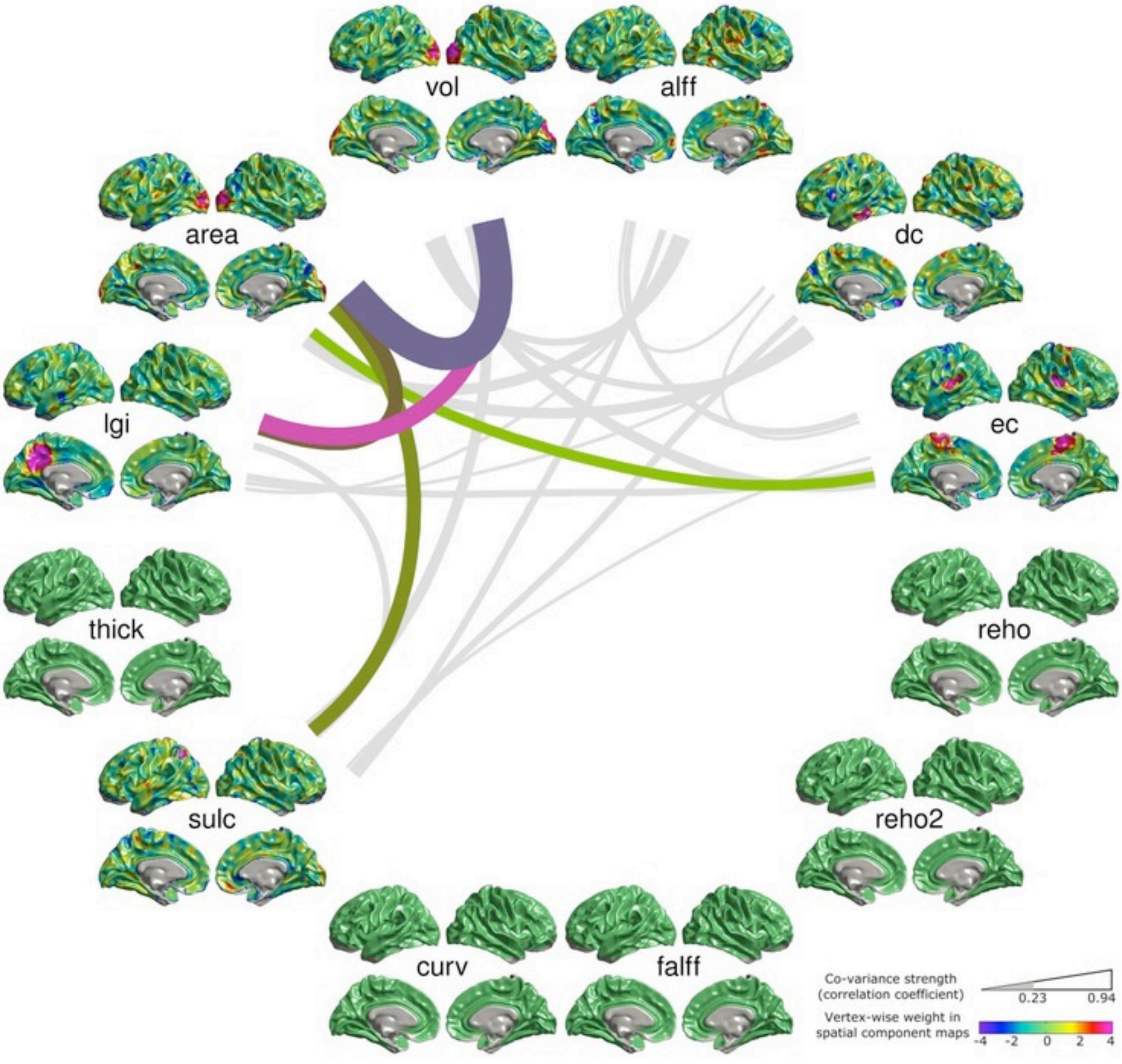

Figure S15. MMCU14, ICC = 0.84, CC = 0.87

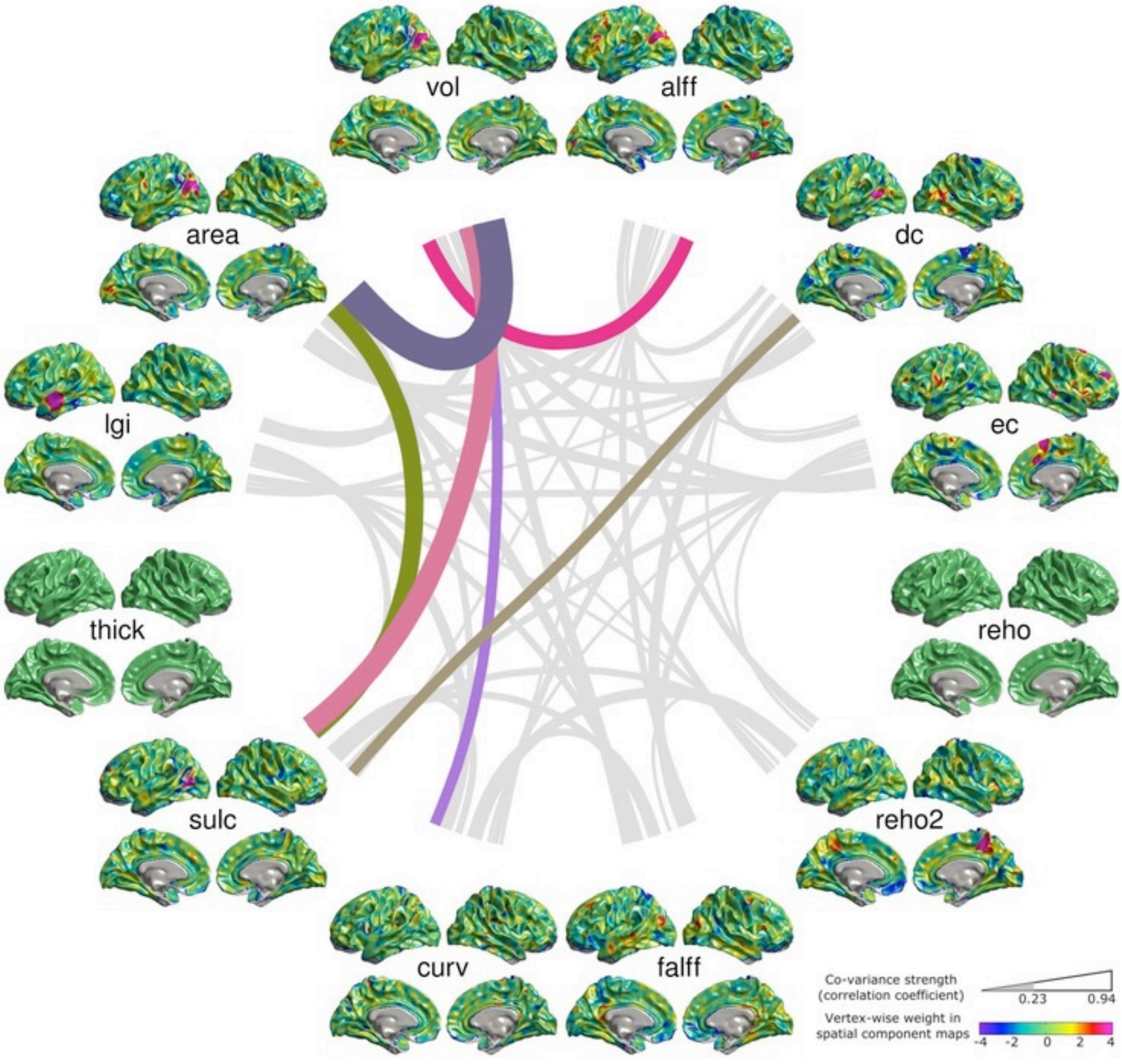

Figure S16. MMCU15, ICC = 0.82, CC = 0.85

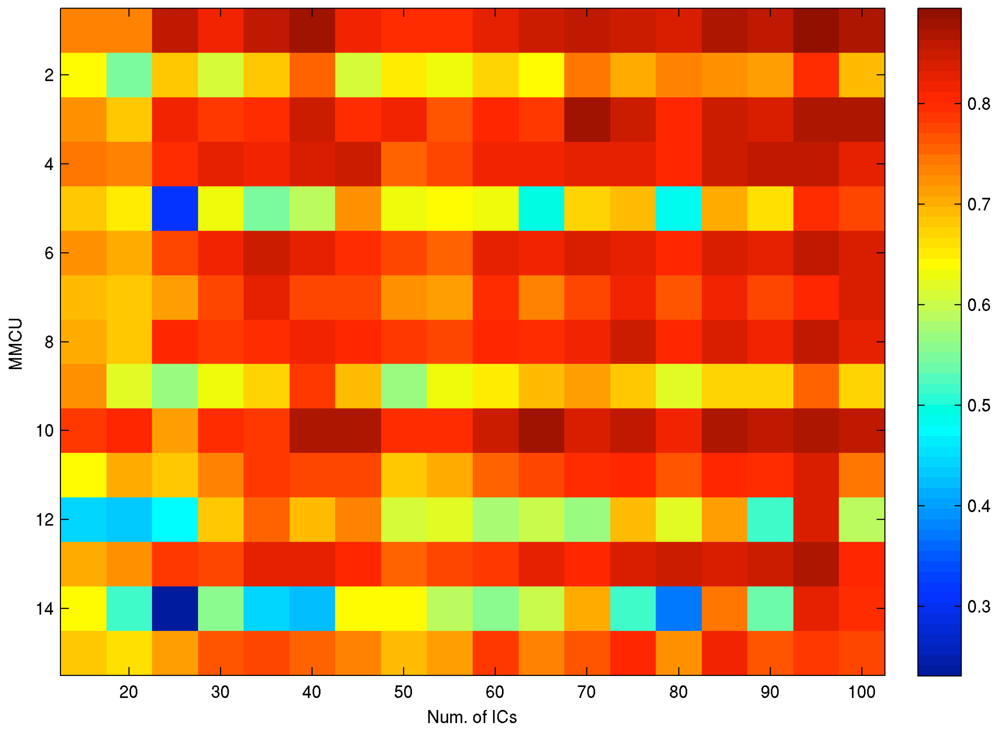

Figure S17. Correlation coefficients between the matched MMCUs when setting different numbers of components in ICA decompositions. The different numbers of components do not dramatically change the similarity across the matched MMCUs.
